# Genome-Wide Identification and Characterization of PIN-FORMED (PIN) Gene Family Reveals Role in Developmental and Various Stress Conditions in *Triticum aestivum* L.

**DOI:** 10.3390/ijms22147396

**Published:** 2021-07-09

**Authors:** Manu Kumar, Bhagwat Singh Kherawat, Prajjal Dey, Debanjana Saha, Anupama Singh, Shashi Kant Bhatia, Gajanan Sampatrao Ghodake, Avinash Ashok Kadam, Hyun-Uk Kim, Manorama, Sang-Min Chung, Mahipal Singh Kesawat

**Affiliations:** 1 Department of Life Science, College of Life Science and Biotechnology, Dongguk University, Goyang 10326, Korea; manukumar007@gmail.com; 2 Krishi Vigyan Kendra, Swami Keshwanand Rajasthan Agricultural University, Bikaner 334603, India; skherawat@gmail.com; 3 Faculty of Agriculture, Sri Sri University, Cuttack 754006, India; prajjal.d@srisriuniversity.edu.in (P.D.); anupama.s@srisriuniversity.edu.in (A.S.); 4 Department of Biotechnology, Centurion University of Technology and Management Jatni, Bhubaneswar 754006, India; debanjana.saha@cutm.ac.in; 5 Department of Biological Engineering, College of Engineering, Konkuk University, Seoul 143701, Korea; shashibiotechhpu@gmail.com; 6 Department of Biological and Environmental Science, Dongguk University, Goyang 10326, Korea; ghodakegs@gmail.com; 7 Research Institute of Biotechnology and Medical Converged Science, Dongguk University, Goyang 10326, Korea; kadamavinash@dongguk.edu; 8 Department of Bioindustry and Bioresource Engineering, Plant Engineering Research Institute, Sejong University, Seoul 05006, Korea; hukim64@sejong.ac.kr; 9 Department of Dairy Microbiology, College of Dairy Science and Food Technology, Raipur 49200, India; manoramachauhan2@gmail.com

**Keywords:** PIN, auxin, qRT-PCR, cis-acting regulatory elements, biotic and abiotic stress, polar auxin transport

## Abstract

PIN-FORMED (PIN) genes play a crucial role in regulating polar auxin distribution in diverse developmental processes, including tropic responses, embryogenesis, tissue differentiation, and organogenesis. However, the role of PIN-mediated auxin transport in various plant species is poorly understood. Currently, no information is available about this gene family in wheat (*Triticum aestivum* L.). In the present investigation, we identified the PIN gene family in wheat to understand the evolution of PIN-mediated auxin transport and its role in various developmental processes and under different biotic and abiotic stress conditions. In this study, we performed genome-wide analysis of the PIN gene family in common wheat and identified 44 TaPIN genes through a homology search, further characterizing them to understand their structure, function, and distribution across various tissues. Phylogenetic analyses led to the classification of TaPIN genes into seven different groups, providing evidence of an evolutionary relationship with *Arabidopsis thaliana* and *Oryza sativa*. A gene exon/intron structure analysis showed a distinct evolutionary path and predicted the possible gene duplication events. Further, the physical and biochemical properties, conserved motifs, chromosomal, subcellular localization, transmembrane domains, and three-dimensional (3D) structure were also examined using various computational approaches. Cis-elements analysis of TaPIN genes showed that TaPIN promoters consist of phytohormone, plant growth and development, and stress-related cis-elements. In addition, expression profile analysis also revealed that the expression patterns of the TaPIN genes were different in different tissues and developmental stages. Several members of the TaPIN family were induced during biotic and abiotic stress. Moreover, the expression patterns of TaPIN genes were verified by qRT-PCR. The qRT-PCR results also show a similar expression with slight variation. Therefore, the outcome of this study provides basic genomic information on the expression of the TaPIN gene family and will pave the way for dissecting the precise role of TaPINs in plant developmental processes and different stress conditions.

## 1. Introduction

The phytohormone auxin plays a key role in plant developmental processes [1,2,3,4]. Auxin forms gradients and concentration maxima in tissues and organs to stimulate diverse biological processes including gravitropism [5,6], organ initiation [7], leaf venation [8], apical dominance [9], embryo axis formation [10], root architecture [11], leaf vascular development [12], tropisms [13,14], fruit ripening [15], phototropism [16], phyllotactic patterning [17], lateral root emergence [18], root hair growth [19], apical hook development and root patterning [20], and sporophyte and male gametophyte development [21,22]. Several researchers have demonstrated that metabolic changes and transport of auxin play a key role in tissue differentiation, embryogenesis, organogenesis, differential growth, and tropic responses [2,3,4,23,24,25,26]. Auxin is synthesized in various plant tissues by several different pathways [27,28] and subjected to long- and short-range transport mediated by influx and efflux auxin transporters [25,29]. Auxin concentration maxima formation is accomplished by polar auxin transport by influx and efflux auxin transport proteins. The polar auxin transport between cells is facilitated by three major auxin transporter families: AUXIN-RESISTANT1 (AUX1)/AUX1-LIKEs proteins for auxin influx [5,30], PIN proteins [25,31], and ATP binding cassette family members for auxin efflux [32,33]. Among these, PIN family proteins play a key role in directional polar auxin transport and formation of local auxin gradients since these PINs are asymmetrically located at the plasma membrane and their polarity regulates the route of intercellular auxin flow in response to different endogenous and environmental signals [33,34,35].

PIN proteins have been well characterized in Arabidopsis, which includes eight members that differ in the length of a middle region [34,36,37]. Five of the Arabidopsis PINs (AtPIN1-4 and AtPIN7) have a long hydrophilic loop situated at the plasma membrane, implicated in the directional and cell-to-cell auxin transport [2,22,38]. Further, these long AtPINs do not localize statically in the plasma membrane; however, they constitutively cycle between the plasma membrane and endosomal compartments, and their relocation is triggered by internal and external stimuli [39], while three AtPINs, namely, AtPIN5, AtPIN6 and AtPIN8, contain a smaller central hydrophilic domain, and both AtPIN5 and AtPIN8 are located in the endoplasmic reticulum, indicating a potential role in modulating intracellular auxin homeostasis [40,41]. Interestingly, AtPIN6 is localized in the endoplasmic reticulum and plasma membrane, indicating that it might be involved in intercellular auxin transport and cellular auxin homeostasis [42]. Furthermore, PIN proteins are highly conserved from primitive to modern plants, although the sizes of their genomes and their numbers of genes differ greatly in plants, for instance, 8 PIN genes in Arabidopsis [36,43], 17 in cotton [44], 14 in maize [45], 10 in pepper [46], 12 in rice [47], 15 in poplar [48], 23 in soybean [49], 10 in tomato [50], 29 in tobacco [51], 11 in sorghum [52], and 10 in potato [53]. Several homologous PIN genes have been characterized in monocots and dicots [45,47,54,55], including the following examples: overexpression of the ABP1 altered PIN-mediated auxin transport in tobacco [56]; NtPIN4 regulates axillary bud growth in tobacco [51]; OsPIN1b regulates seminal root elongation in response to low phosphate and nitrogen in rice [57]; and MtPIN1 and MtPIN3 regulate the shade avoidance response under various environments in *Medicago truncatula* [58].

With recent advances in sequencing technologies, there has been a dramatic increase in the number of sequenced plant genomes in recent years. Although genome sequence databases have provided researchers with a wealth of encoded information, the genes identified in plant species’ genomes are still uncharacterized, particularly in terms of their function and regulation. The structural and functional characterization of those genes is now a challenging approach [59]. Common wheat (*Triticum aestivum* L.) is a major cereal and staple crop worldwide, providing food for humans and feed for animals [60,61]. Wheat is an important source of carbohydrates, protein, vitamins, and minerals for humans [61,62,63,64]. However, wheat production is adversely affected by several biotic and abiotic stresses such as insects, fungal, bacterial, and viral diseases, heat, drought, cold, and salinity [63,65,66]. Therefore, several researchers have concentrated on improving productivity, quality, and stress tolerance in wheat. Wheat is originated from the natural hybridization of three closely related genomes: A, B and D. The wheat genome has already been sequenced completely, and 124,201 genes have been identified [61]. Gene distribution analysis across the three subgenomes revealed that there is a higher number of genes on the B subgenome with 44,523 (35%), compared to the A and D subgenomes, which had 40,253 (33%) and 39,425 (32%), respectively [61]. In the present investigation, we identified the PIN gene family in wheat to understand the evolution of PIN-mediated auxin transport and its role in various developmental processes and under different biotic and abiotic stress conditions. In this study, we performed genome-wide analysis of the PIN gene family in common wheat and identified 44 TaPIN genes using various computational approaches, further characterizing them to understand their structure, function, and distribution across various tissues.

## 2. Results

### 2.1. Identification of PIN Family Members in T. aestivum

A total of 44 PIN genes were identified in the wheat genome (Table 1); this number is relatively high compared to the previously reported PINs in other plant species such as Arabidopsis, rice, sorghum, and maize (Table 2). 

This result might be owing to the higher ploidy level and large genome size of wheat. Bread wheat is originated from the natural hybridization of three closely related genomes (A, B and D) [67]. The TaPIN family had an average molecular weight of 49.87 kDa. The average isoelectric point (pI) of the TaPIN family ranged from 5.92 to 9.32; TaPIN44 had the highest pI, 9.32, while TaPIN38 had the lowest pI of 5.92. We also plotted the molecular weight of TaPINs with their pI to examine the molecular weight distribution of different TaPIN family members (Figure S1). The plots indicate that most TaPINs have a similar molecular weight and pI, and they clustered together. The calculated grand average of hydropathy index (GRAVY) values of all TaPINs was 0.011 to 0.809, indicating that they were hydrophobic in nature. The determination of the subcellular localization of TaPIN proteins will help to understand the molecular function. The subcellular localization prediction of TaPIN proteins suggests that most TaPINs were located on the plasma membrane (Table 1).

**Figure 1:**
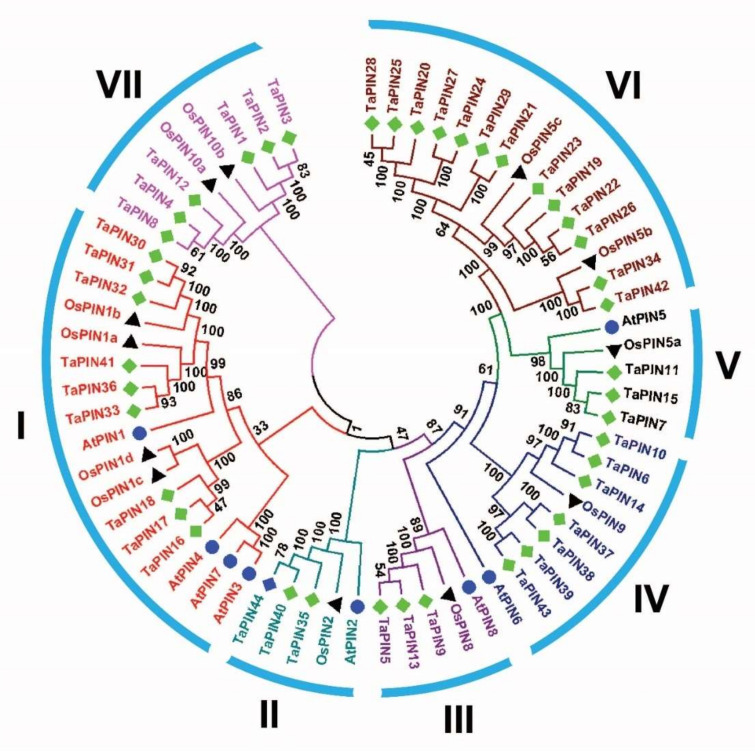
Phylogenetic analysis of PIN proteins among wheat (44), Arabidopsis (8), and rice (12) using MEGAX by the neighbor joining method.

To investigate the evolutionary relationship between TaPINs and other plant species PINs, a phylogenetic tree was constructed with TaPIN, AtPIN and OsPIN proteins (Table S1). The results indicate that TaPIN proteins were divided into seven groups (Figure 1), where group VI was the largest with 13 members. Groups I, II, III, IV, V and VII included nine, three, three, seven, three and six members, respectively (Figure 1 and Figure S2).

### 2.2. Chromosomal Distribution of TaPIN Genes

**Figure 2:**
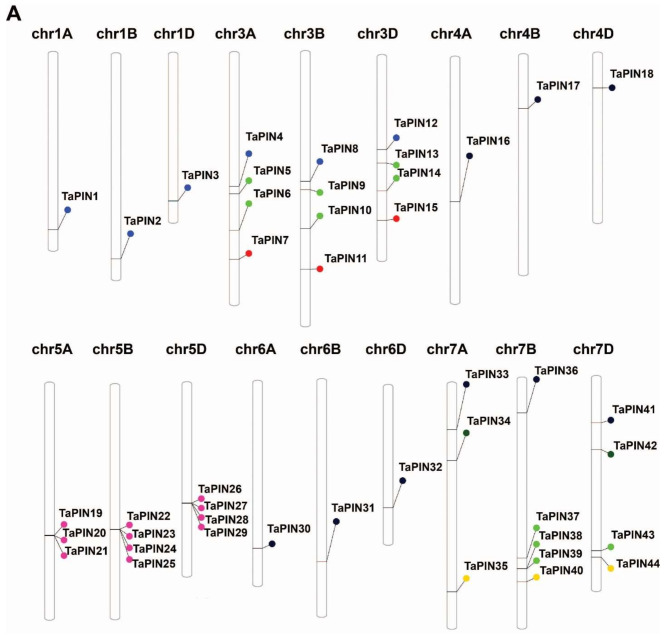

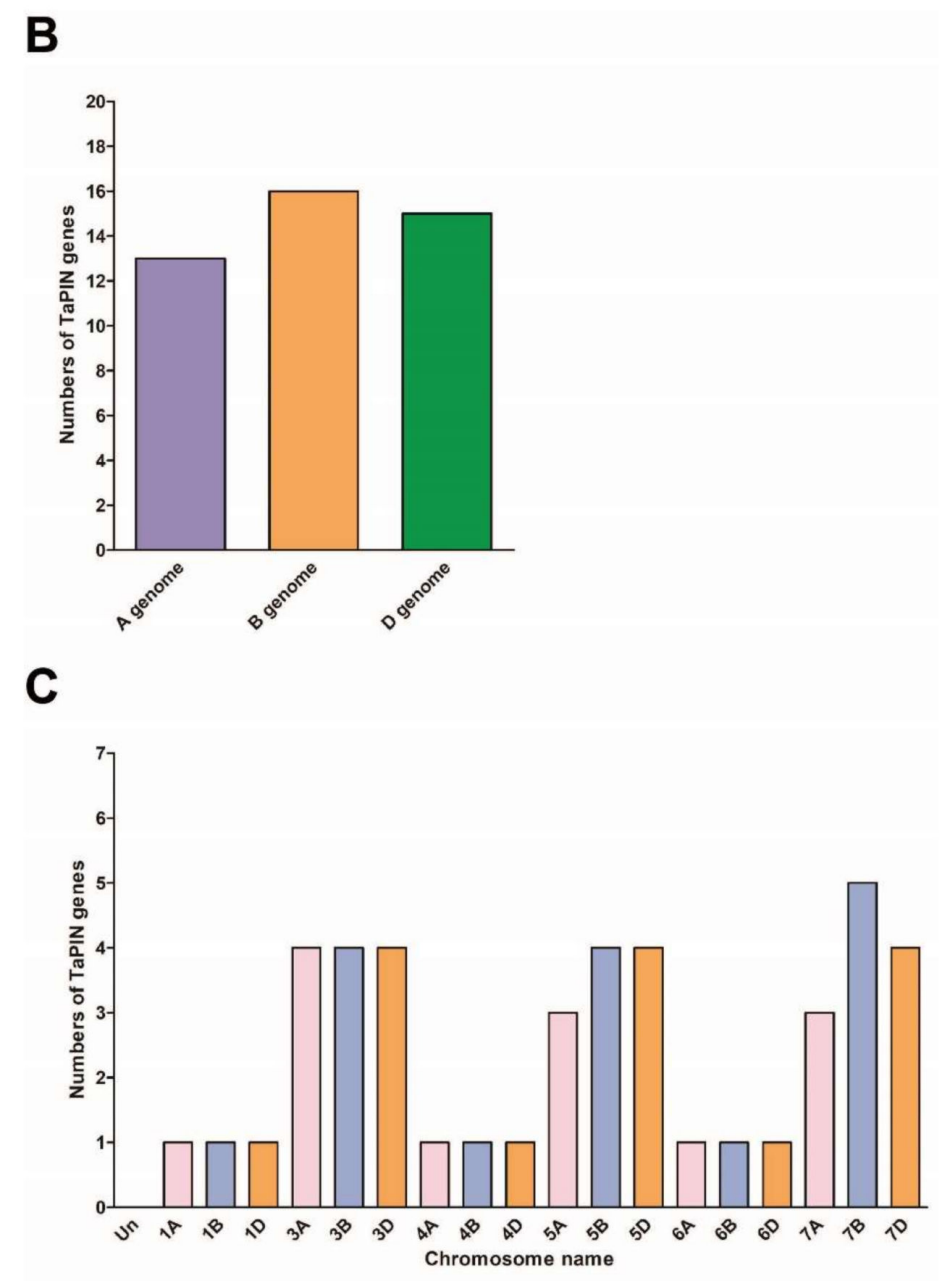
Genomic distribution of identified PIN genes on the 21 chromosomes of wheat and within the three subgenomes. (**A**) Schematic representations of the chromosomal distribution of PIN genes on the 21 chromosomes of wheat and the name of the gene on the right side. The colored round circle on the chromosomes indicates the position of the PIN genes. The chromosome numbers of the three subgenomes are indicated at the top of each bar. (**B**) Distribution of PIN genes in the three subgenomes. (**C**) Distribution of PIN genes across 21 chromosomes.

The genomic chromosomal distribution of the identified TaPIN genes in wheat was mapped to the corresponding chromosomes according to the chromosomal locations of PIN genes by the PhenGram online server. The TaPIN genes are present on the 18 wheat chromosomes (Figure 2A and Table 1). TaPIN genes displayed a slightly higher presence on the B and D subgenomes (Figure 2B). The maximum number of TaPIN genes, for instance, 16, was mapped on the chromosomes of the B subgenome. A subgenome had the lowest number of TaPIN genes, i.e., 13. The maximum number of TaPINs is located on chromosome 7B with five genes (Figure 2C). The minimum number of TaPINs is located on chromosome 1A, 1B, 1D, 4A, 4B, 4D, 6A, 6B and 6D, having only a single gene. Conversely, none of the TaPIN genes were found on chromosome 2. Overall, all the PIN family genes were evenly distributed on the three subgenomes of wheat. 

In the context that wheat is hexapolyploid with large genomes, we further examined the duplication events in the TaPIN gene family. The phylogenetic analysis of the TaPIN genes also indicates several duplication events (Figure S3). We found that 30 PIN genes in *T. aestivum* participated in duplication events (Figure S4 and Table S2), which points out that the expansion of the PIN gene family in *T. aestivum* was caused mainly by whole-genome duplication or segmental duplication within genomes. To elucidate the selective pressure on the duplicated TaPIN genes, we calculated the non-synonymous (Ka) and synonymous substitutions (Ks), and the Ka/Ks ratios for the 15 TaPIN gene pairs (Table S2). The value of Ka/Ks = 1 denotes that genes experienced a neutral selection; <1 suggests a purifying or negative selection; and >1 indicates a positive selection [68]. The Ka/Ks values for all 15 gene pairs were less than 1, suggesting that TaPIN genes underwent a strong purifying or negative selection pressure, with slight changes after duplication. Thus, these results indicate the conserved evolution of TaPIN genes.

**Figure 3:**
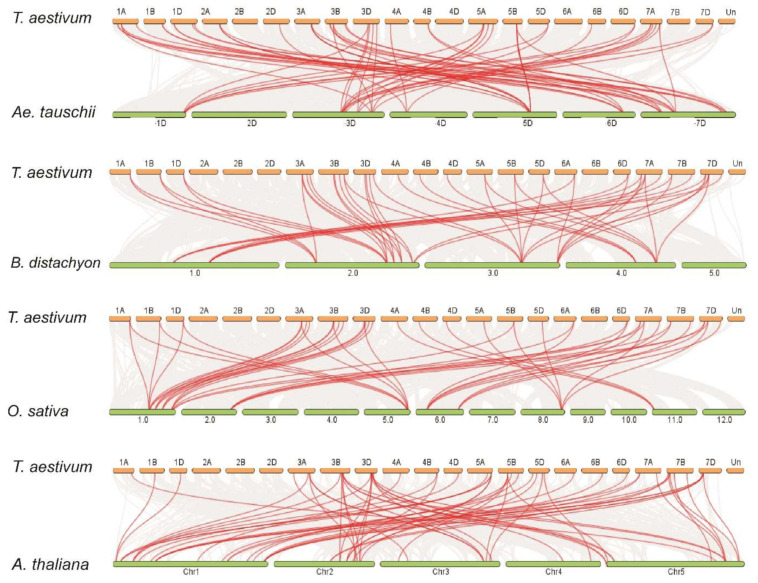
Syntenic relationships of TaPIN genes between *Aegilops tauschii*, *Brachypodium distachyon*, *Oryza sativa* and *Arabidopsis thaliana*. The gray lines in the background represent the collinear blocks within *Triticum aestivum* and other plant genomes, while the red lines highlight the syntenic PIN gene pairs.

To further examine the synteny relationships of TaPIN genes with other wheat relatives and model plants such as *B. distachyon, Ae. tauschii*, *T. dicoccoides*, *O. sativa* and *A. thaliana*, the Multiple Collinearity Scan toolkit was used to find the orthologous genes between these plant species’ genomes (Figure 3 and Table S3). We identified 38, 31, 68, 40 and 36 orthologous gene pairs between TaPINs with other PIN genes in *B. distachyon, Ae. tauschii*, *T. dicoccoides*, *O. sativa* and *A. thaliana*, respectively. The results show that 30, 25, 42, 36 and 24 TaPIN genes were collinear with PIN genes in *B. distachyon*, *Ae. tauschii*, *T. dicoccoides*, *O. sativa* and *A. thaliana*, respectively. A few TaPIN genes had at least three pairs of orthologous genes, for instance, *TaPIN1, TaPIN2, TaPIN3, TaPIN4, TaPIN8* and *TaPIN12*, which might have played a crucial role in the evolution of PIN genes. These results suggest that TaPIN genes in wheat might be originated from other plant species’ orthologous genes.

### 2.3. Gene Structure Analysis of TaPIN Genes

**Figure 4:**
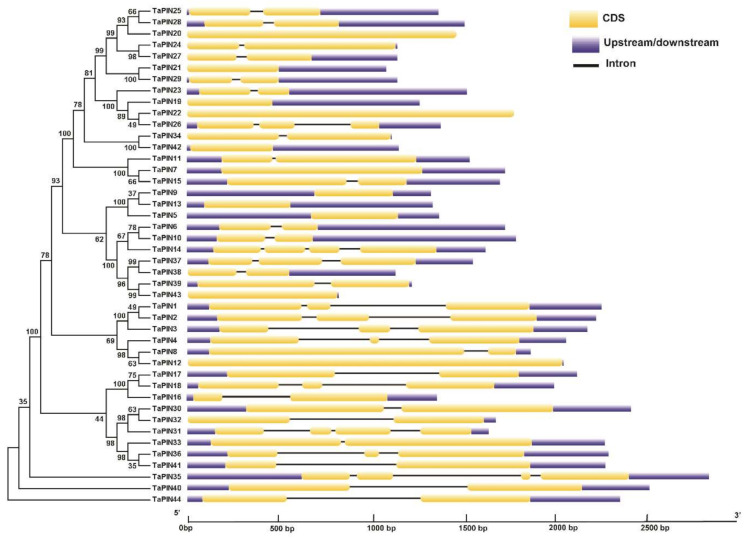
Exon–intron organization of the TaPIN genes. Yellow boxes represent exons, untranslated regions (UTRs) are indicated by blue boxes, and black lines represent introns. The lengths of the boxes and lines are scaled based on gene length. The exon and intron sizes can be estimated using the scale at the bottom.

**Figure 5:**
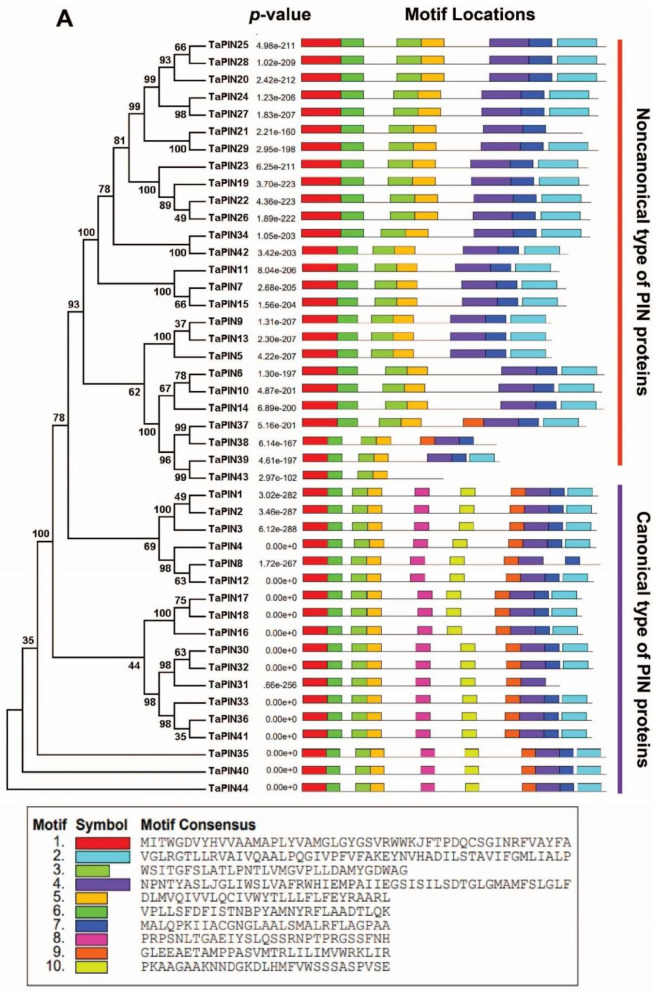

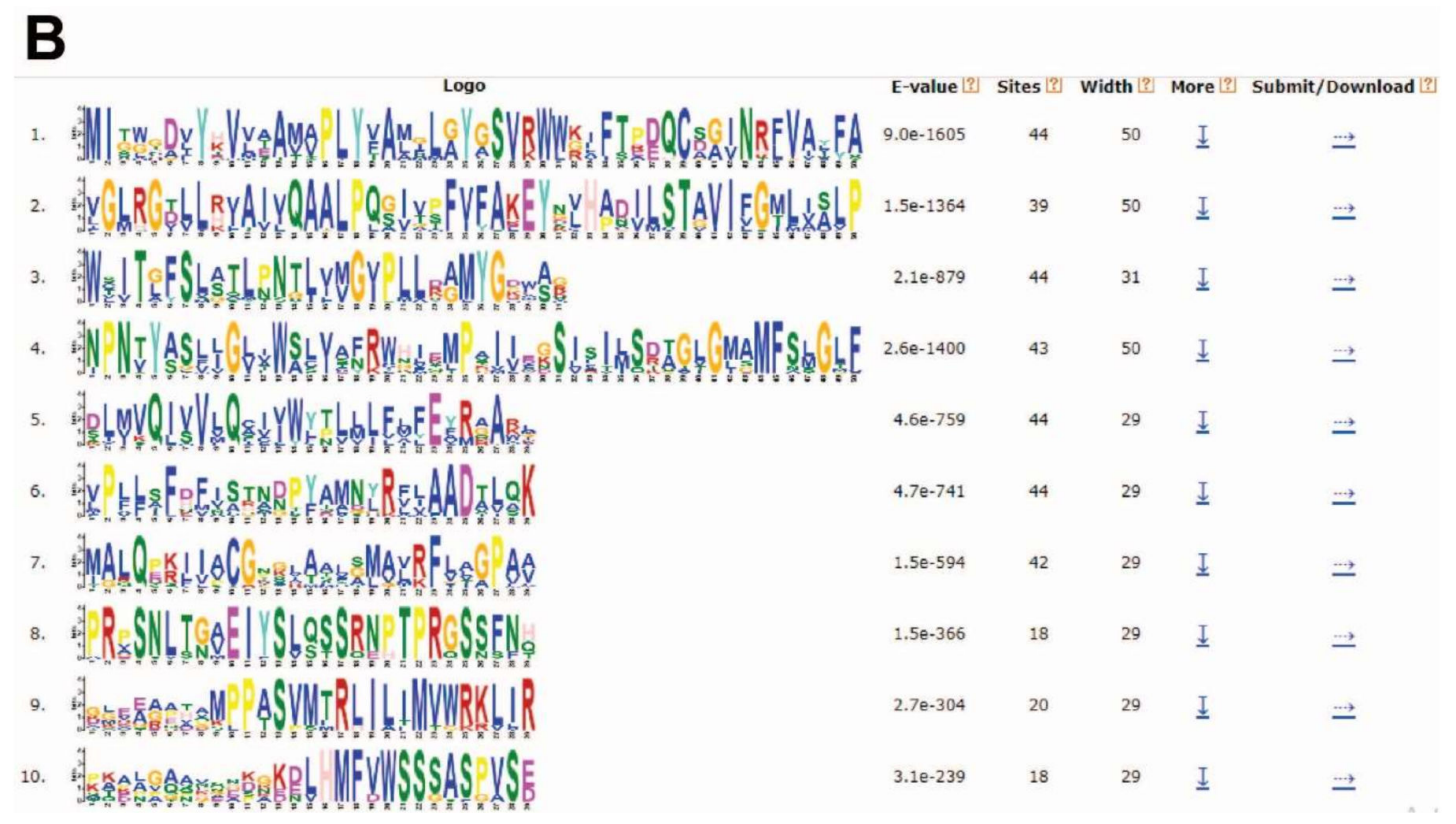
Conserved motifs of TaPIN genes elucidated by MEME. (**A**) Colored boxes representing different conserved motifs having different sequences and sizes. (**B**) Sequence logo conserved motif of the wheat PIN proteins. The overall height of each stack represents the degree of conservation at this position, while the height of the individual letters within each stack indicates the relative frequency of the corresponding amino acids.

To understand the structural characteristics of the TaPIN genes, the exon–intron structures (Figure 4) and conserved motifs (Figure 5A,B) of TaPIN genes were analyzed. Gene structure analysis revealed that the TaPIN gene family varied greatly in terms of gene structure as most of the PIN genes contain one–three introns; however, some members of the TaPIN gene family are intronless such as *TaPIN5*, *TaPIN7*, *TaPIN9*, *TaPIN12, TaPIN13*, *TaPIN19-21*, *TaPIN42* and *TaPIN43*. A maximum of three introns were found in *TaPIN14* and *TaPIN31* (Figure S5).

### 2.4. Conserved Motif Analysis of TaPIN Genes

**Figure 6:**
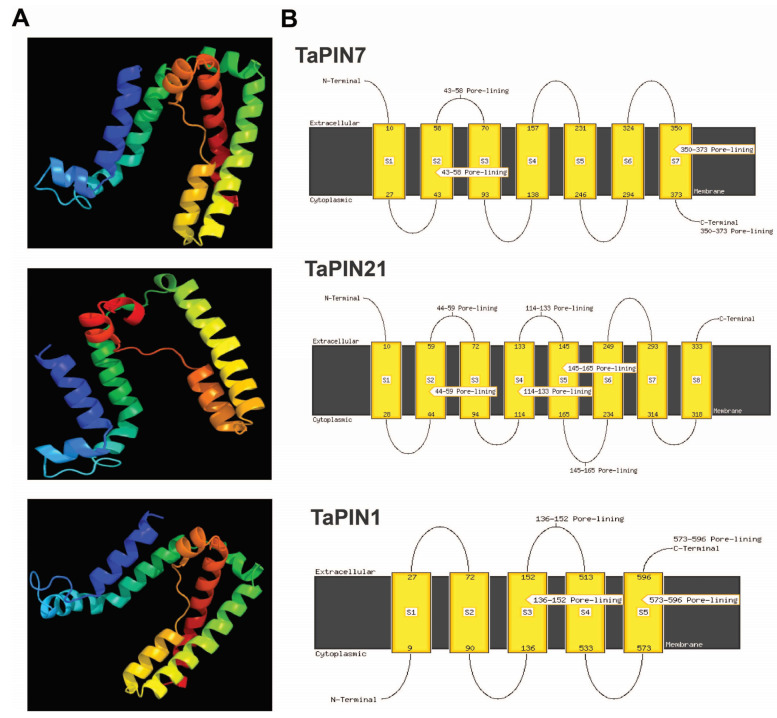

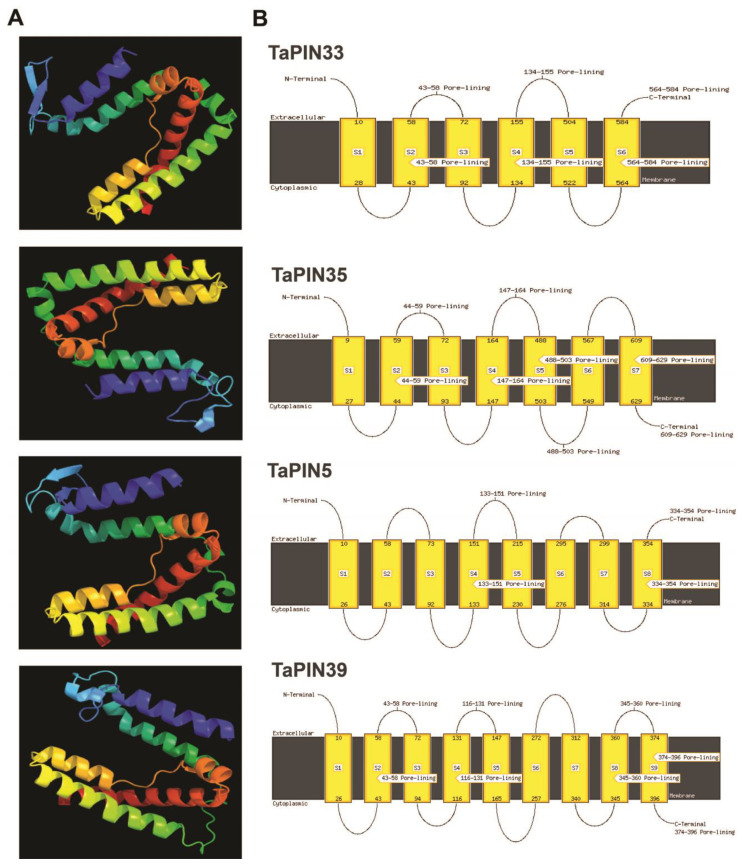
Predicted 3D structures and transmembrane helix of seven selected TaPIN proteins. (**A**) Three-dimensional structure and (**B**) TM helix of seven TaPINs representing each group of a phylogenetic tree. The cytoplasmic and extracellular sides of the membrane are labeled, and the start and end of each transmembrane helix are indicated with a number.

Further, we also elucidated the conserved motifs of TaPIN genes using the MEME (Multiple Em for Motif Elicitation) online servers. Finally, 10 conserved motifs were identified in 44 TaPIN genes (Figure 5A,B). The TaPIN gene family was identified by the presence of a membrane transport domain (Pfam 03547), and all TaPINs had at least one membrane transport domain (Table S4) involved in auxin efflux, maintaining auxin homeostasis. In order to understand the molecular function of TaPIN genes in *T. aestivum*, three-dimensional (3D) protein models and transmembrane helices of all TaPINs were generated using the phyre2 server. TaPIN 3D protein structures contained α-helical bundles forming five to nine transmembrane helices (H1 to H9) (Figure 6A). The predicted transmembrane helices of the TaPIN proteins displayed a similar structure: a conserved amino and carboxy-terminal region of transmembrane segments and a divergent central region supposed to be a cytoplasmic domain (Figure S6), except for TaPIN5, TaPIN6, TaPIN7, TaPIN9, TaPIN10, TaPIN11, TaPIN13, TaPIN14, TaPIN15, TaPIN19-29, TaPIN34, TaPIN37, TaPIN38, TaPIN39, TaPIN42 and TaPIN43, which had a short central loop. In addition, multiple sequence alignment also revealed that all TaPIN proteins share a highly conserved N- and C-terminal and a variable central region (Figure 6B, Figures S6 and S7). These results will help to understand the substrate specificity and molecular function of TaPIN genes.

### 2.5. Putative Cis-acting Regulatory Elements (CAREs) Analysis of TaPIN Genes

**Figure 7:**
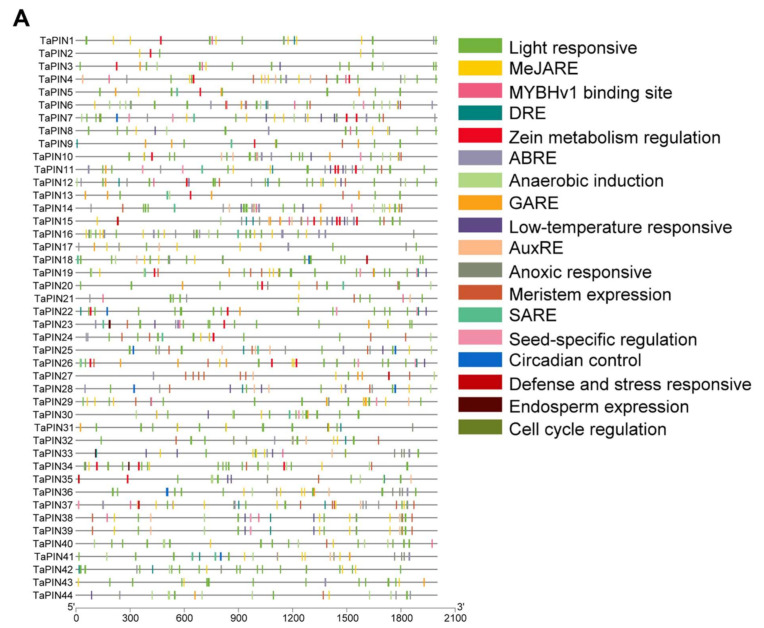

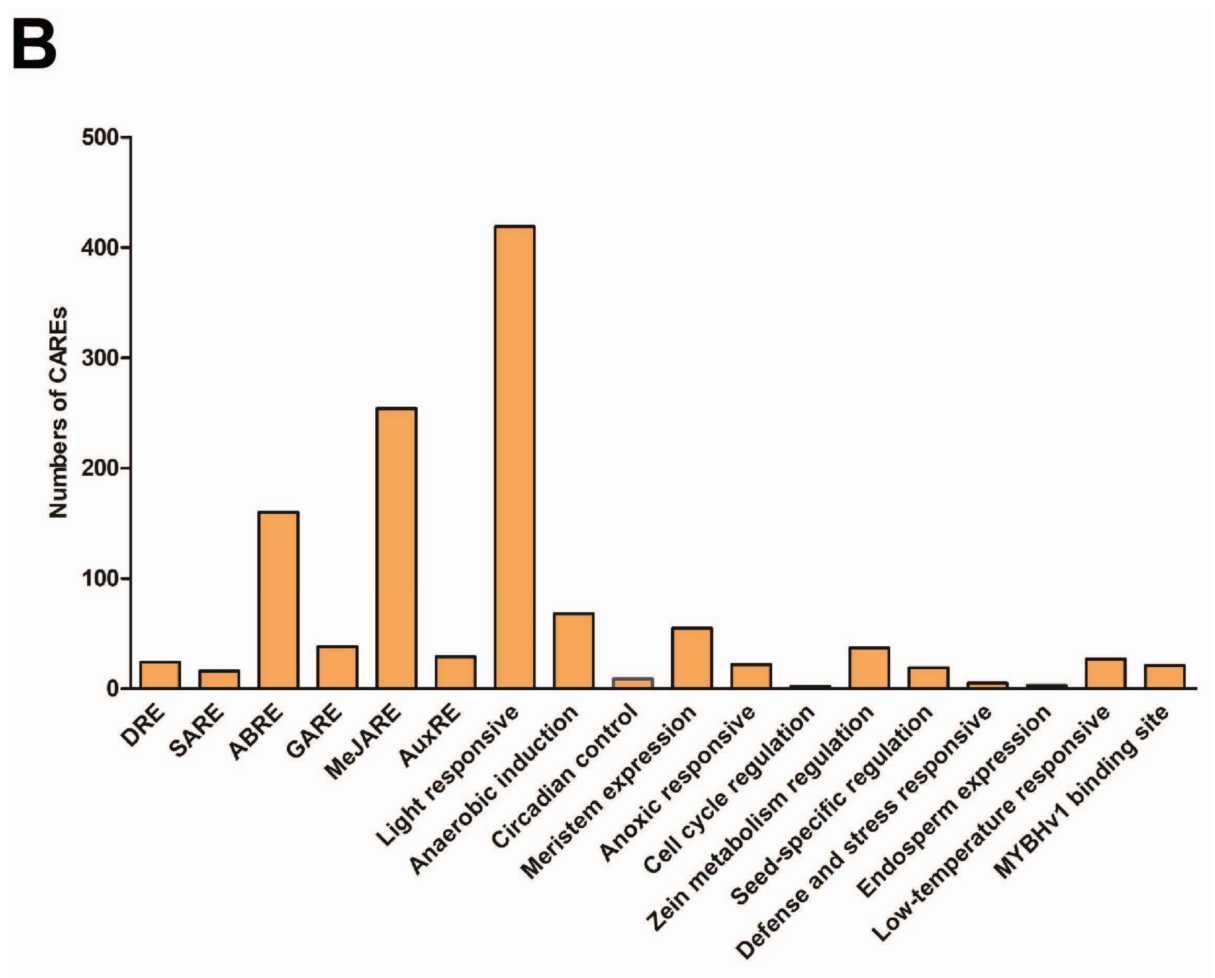
Putative cis-acting regulatory elements (CAREs) of the TaPIN gene family. The CAREs analysis was performed with the 2 kb upstream region using the PlantCARE online server. (**A**) Hormone-responsive elements, stress-responsive elements, growth and development-related elements, light-responsive elements, and other elements with unknown functions are shown by different colors. (**B**) Most commonly occurring CAREs in TaPINs.

To further understand the potential regulatory mechanism of TaPIN genes, and how these genes are regulated by phytohormone, various defense, and stress-responsive elements, the PlantCARE webserver was employed to find out putative cis-elements in the 2000 bp promoter region of TaPINs. A total of 18 unique CAREs were identified in the TaPIN gene family, including elements related to light responses, methyl jasmonate (MeJA), abscisic acid response, auxin response, salicylic acid response, defense, and stress responses (Figure 7A and Table S5). CAREs involved in light, MeJA, auxin, and abscisic acid responses were the most prevalent ones in the TaPIN gene family (Figure 7B). This suggests that TaPINs play an essential role in plant growth and developmental processes. Further, light-responsive CAREs were also abundant in the TaPIN gene family. These CAREs have been implicated in photosynthesis/non-photosynthesis-based light responses and circadian rhythm-mediated light responses.

TaPINs also had CAREs related to meristem expression and seed-specific regulation. The CAREs present in the TaPIN gene family suggest that they have been involved in diverse developmental processes that can be regulated by hormones, light, and various developmental stages. The presence of multiple putative CAREs in the TaPIN promoters indicates that these genes might be involved in a wide range of biological processes. Thus, these data provide valuable insights to understand the TaPIN gene family’s response to different stress, phytohormone, and other developmental processes.

### 2.6. Gene Ontology (GO) Enrichment of TaPIN Genes

Gene ontology helps to understand the function of genes by examining their similarity with other species’ genes of known function. All TaPINs were effectively annotated and assigned GO terms using AgriGO (Figure S8 and Table S6). TaPINs were also annotated using eggNOG-Mapper for further confirmation (Table S7), which returned similar results to AgriGO. In the biological process category, TaPIN genes are enriched in the signaling (GO:0023052) and meristem maintenance (GO:0010073) categories (Figure S8A). In the cellular component category, TaPINs showed enrichment in the membrane (GO:0016020) (Figure S8B). The prediction of subcellular localization was carried out by CELLO, and BUSCO (Table 1) also returned similar results. In the molecular function category, transporter activity (GO:0005215) was the most enriched category mainly involved in auxin transport activity (Figure S8C). Apart from signaling and meristem maintenance, the GO term enrichment also indicated multiple roles of TaPIN genes, including transport of auxin, ions, response to gravity, endogenous stimuli, tropism, embryonic specification pattern, axis specification, leaf formation, floral organ development, determination of bilateral symmetry, immune responses, and metabolism. Therefore, these results suggest that TaPIN genes play a crucial role in plant developmental processes.

### 2.7. Expression Profiling of TaPIN Genes under Various Stress Conditions and Developmental Stages

**Figure 8:**
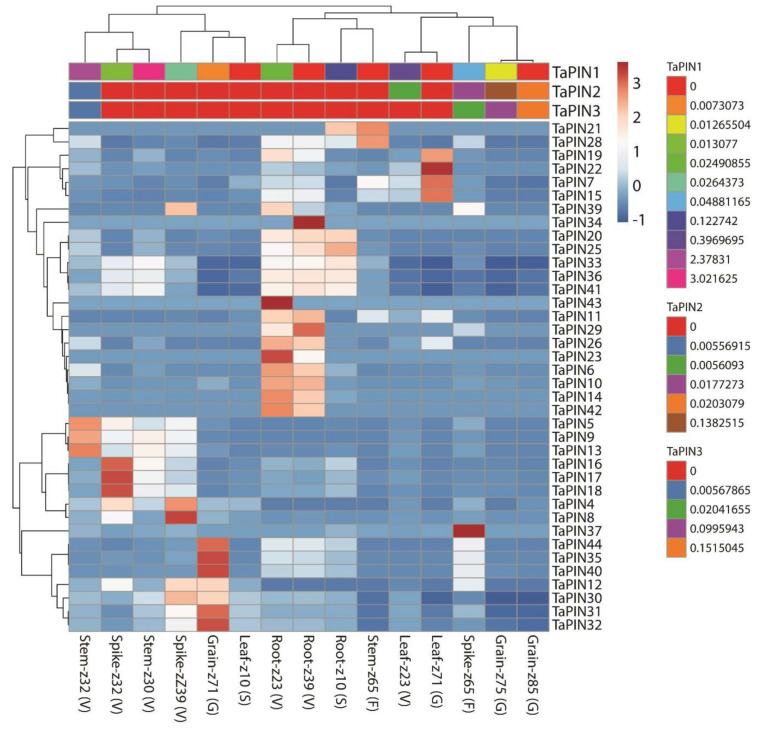
Heatmap representing the expression pattern of TaPIN genes in various developmental stages. TPM values were directly used to create the heatmap.

**Figure 9:**
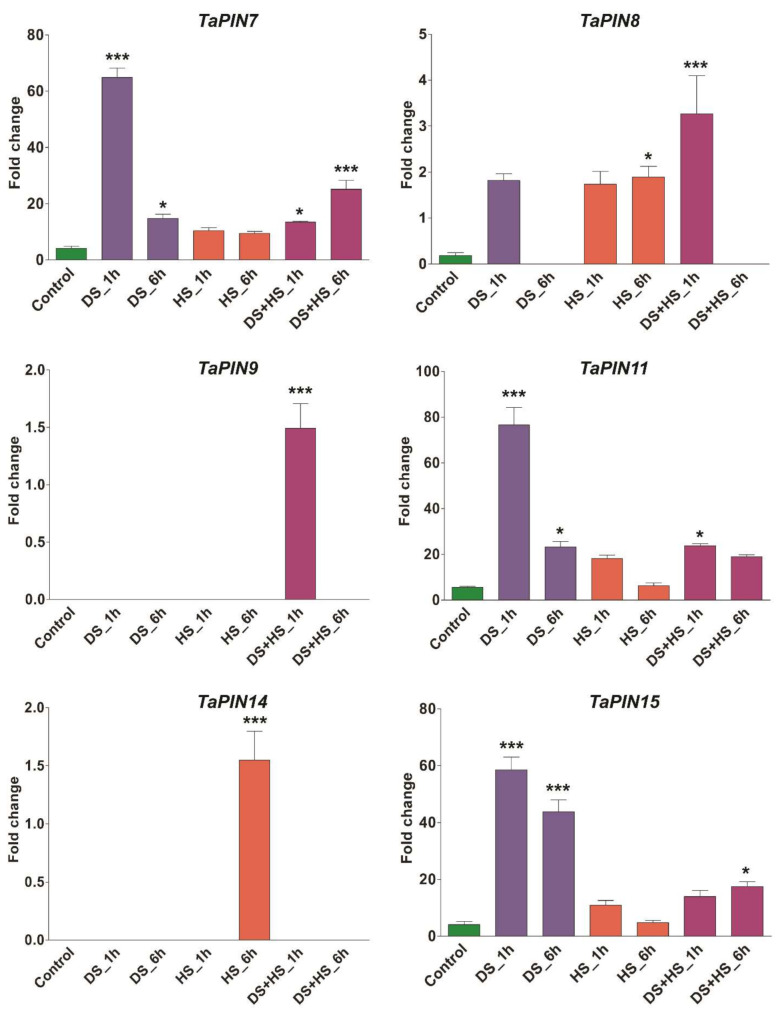

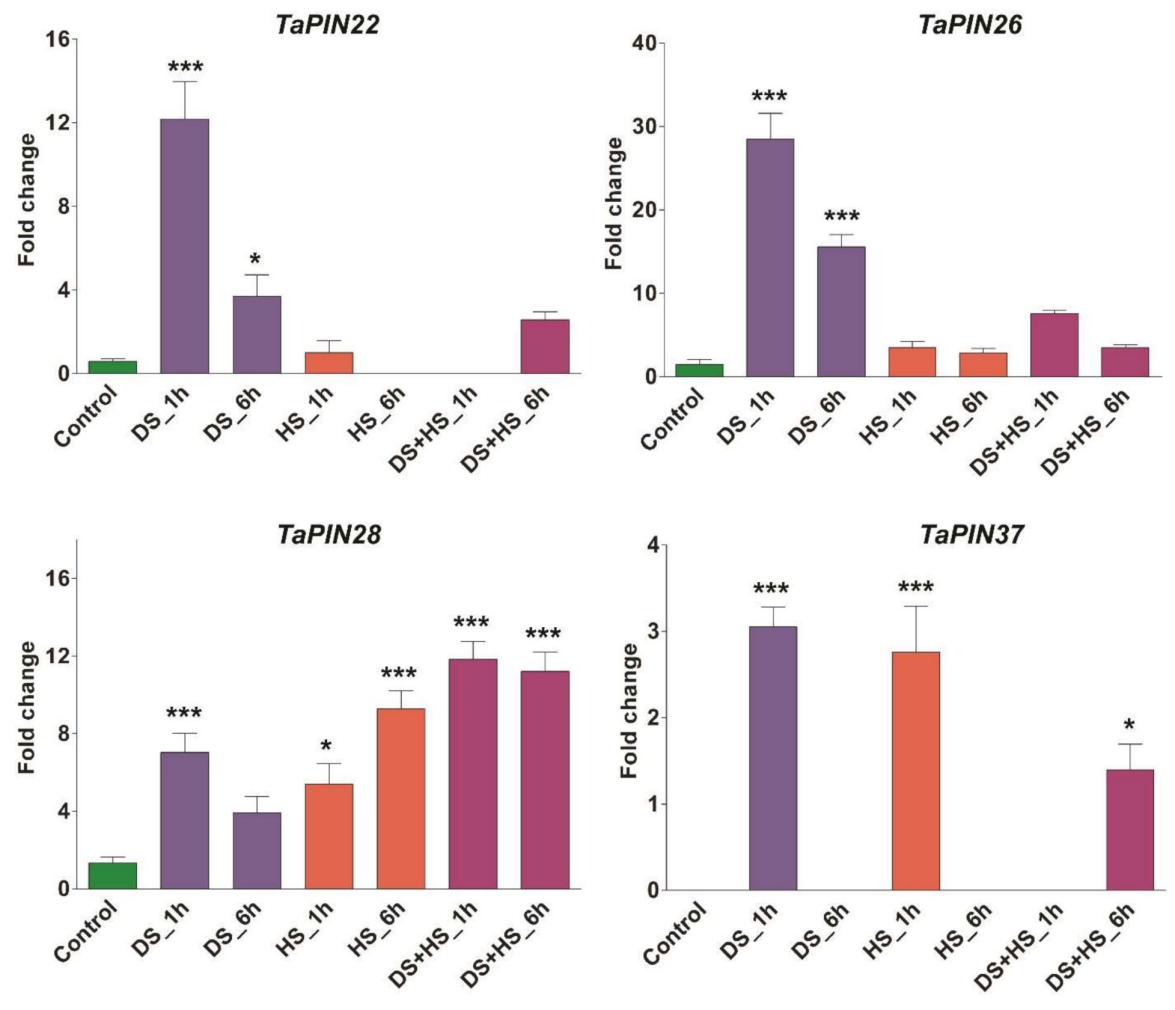
Quantitative real-time PCR analysis of selected *TaPIN* genes in response to drought and heat stress to verify RNA seq data. The wheat actin gene was used as the internal control to standardize the RNA samples for each reaction. Asterisk indicates significant differences compared with control over bars representing results of Tukey HSD test at the <0.05 and <0.001 levels (* *p* < 0.05 and *** *p* < 0.001). Error bars show standard deviation. Data are mean ± SD (*n* = 3).

To understand the expression pattern of TaPIN genes in various development stages and different stress conditions, we retrieved transcripts per million (TPM) values of all TaPINs from the wheat expression database. These TPM values were used to produce the principal component analysis (PCA) plot and heatmaps (Figure 8 and Figure 9). Five different tissues from the three different developmental time points were taken to determine the expression profiling of TaPINs in this study. The time points are represented on the Zadoks scale. Different TaPIN genes exhibited differential induction in the different tissues; for instance, *TaPIN4*, *TaPIN8*, *TaPIN12*, *TaPIN30* and *TaPIN39* displayed induction at the spike z39 stage, while *TaPIN16*, *TaPIN17*, *TaPIN18* and *TaPIN37* displayed induction at the spike z32 and spike z65 stages, respectively (Figure 8). The expression of *TaPIN6*, *TaPIN10*, *TaPIN11*, *TaPIN14*, *TaPIN19*, *TaPIN23*, *TaPIN26*, *TaPIN39*, *TaPIN42* and *TaPIN43* was elevated in roots at the z23 stage. *TaPIN6*, *TaPIN10*, *TaPIN11*, *TaPIN14*, *TaPIN26*, *TaPIN29*, *TaPIN34* and *TaPIN42* were also up-regulated in roots at the z39 stage. *TaPIN7*, *TaPIN15*, *TaPIN19* and *TaPIN22* showed induction at the leaf z71 stage. 

In addition, *TaPIN31*, *TaPIN32*, *TaPIN35*, *TaPIN40* and *TaPIN44* expression was raised at the grain z71 stage. *TaPIN5*, *TaPIN9*, *TaPIN13*, *TaPIN21* and *TaPIN28* showed higher expression at stem z32 and z65 stages. These results demonstrate that different TaPINs might participate in the development of different tissues at different stages.

Expression profiling of TaPINs was also examined under three different stress conditions such as biotic (Septoria tritici blotch and powdery mildew) and abiotic stress (drought and heat stress). Several members of the TaPIN family were observed to be induced during biotic and abiotic stress (Figure S10). *TaPIN5*, *TaPIN6* and *TaPIN10* were highly induced upon Septoria infection, while the expression of *TaPIN13*, *TaPIN18*, *TaPIN19*, *TaPIN20*, *TaPIN25*, *TaPIN35* and *TaPIN44* was significantly elevated during powdery mildew infection. In the case of abiotic stress, the data indicate that the expression of some members of the TaPIN family such as *TaPIN7*, *TaPIN11*, *TaPIN15*, *TaPIN22* and *TaPIN26* was induced during the initial hours of drought stress. It seems that the TaPIN family is not involved in heat stress during the initial and late hours; only two TaPIN genes named *TaPIN37* (1 h) and *TaPIN14* (6 h) were induced during heat stress. However, the TaPIN8, TaPIN9 and TaPIN28 genes were up-regulated during the combined heat and drought stress (Figure S10). Expression patterns of some selected TaPIN genes were also verified by qRT-PCR. The qRT-PCR results show almost similar expression trends with slight variation (Figure 9). Overall, the expression pattern was consistent across the two different approaches. Collectively, these results demonstrate that TaPIN family members were involved in the response to drought and heat stress.

### 2.8. Protein–Protein Network Analysis of the TaPIN Family Genes

**Figure 10:**
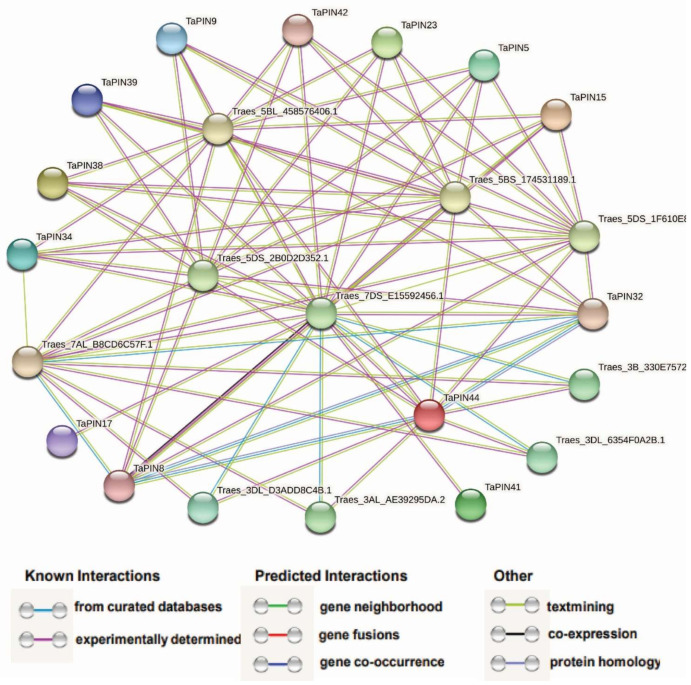
Protein–protein interaction analysis of TaPINs proteins. Protein–protein interaction network produced by STRINGV9.1, where each node represents a protein and each edge represents an interaction, colored by evidence type.

A network was constructed using the STRING database to investigate protein–protein interactions between TaPINs and other wheat proteins (Figure 10 and Table S8).

According to the predicted results, we identified thirteen TaPINs interacting with ten different *T. aestivum* proteins. TaPIN32 can interact with TaPIN8, TaPIN44, and six other *T. aestivum* proteins (Traes_5BL_458576406.1, Traes_5BS_174531189.1, Traes_5DS_1F610E80B.1, Traes_5DS_2B0D2D352.1, Traes_7DS_E15592456.1 and Traes_7AL_B8CD6C57F.1), which were ribosomal protein S6 kinase and protein kinase PINOID, a positive regulator of polar auxin transport through changes in the phosphorylation status of PIN proteins. Meanwhile, TaPIN44 can interact with TaPIN8 and nine other *T. aestivum* proteins (Traes_3AL_AE39295DA.2, Traes_3B_330E75723.1, Traes_3DL_6354F0A2B.1, Traes_3DL_D3ADD8C4B.1, Traes_5BL_458576406.1, Traes_5BS_174531189.1, Traes_5DS_1F610E80B.1, Traes_5DS_2B0D2D352.1 and Traes_7DS_E15592456.1), which were sorting nexin (SNX), ribosomal protein S6 kinase, protein kinase PINOID, and ABC transporter proteins. The sorting nexin (SNX) comprises a large group of proteins that are localized in the cytoplasm, which regulate intracellular trafficking through the lipid binding domain and protein–protein interactions with membrane-associated protein complexes. These results provided valuable information for the further functional characterization of TaPIN genes.

## 3. Discussion

### 3.1. Identification and Evolution of TaPIN Gene Family in Wheat

PINs are plant-specific transmembrane proteins involved in polar auxin transport [4,25,26,34]. PIN family proteins regulate multiple developmental processes, including morphogenesis, embryogenesis, organogenesis, and environmental stimuli [14,22,34,36,53,69,70]. PIN gene family members have been reported in several plant species [44,45,47,48,51,52,53]; however, to the best of our knowledge, this is the first time we identified the PIN genes in the wheat genome. Several studies demonstrated that PIN genes also existed in primitive land plants and expanded over the time of evolution [35,43]—a total of 44 TaPIN genes identified in the wheat genome (Table 1). The phylogenetic analysis revealed that the TaPIN gene family could be classified into seven groups or subfamilies. The phylogenetic analysis showed that the long or canonical PINs were clustered into groups I and II, while short or noncanonical PINs clustered into groups III, V, and VI, and groups IV, VI, and VII consisted of monocot-specific TaPINs (Figure 1). These genes possibly have the monocot-specific functions that might impact physiological and morphological establishment, such as phyllotaxy or development of the unique root system [71], although TaPIN genes were well allocated into the known group of Arabidopsis and rice PINs, suggesting that TaPINs might originate from a common ancestor. Further, the majority of the TaPINs exhibited orthologous relationships with Arabidopsis and rice PINs. The phylogenetic tree also showed that all groups have an expanded number of members (Figure 1), indicating that duplications of these TaPINS occurred during evolution.

Interestingly, none of the TaPIN family members were found with AtPIN6. Similar results were reported in rice and maize [45,47]. Thus, these results demonstrate that the short or noncanonical PINs evolved independently from the long or canonical PINs [43]. The overall evolution pattern showed lineage-specific expansion of the TaPIN gene family through the partial modification of the genome that might reinforce the adaption to internal and external stimuli [72,73].

The wheat PIN gene family is extensively expanded and had relatively more PINs compared to the previously reported PINs in *A. thaliana,* *O. sativa*, *Z. mays*, *G. max* and *N. tabacum* [36,47,49,51,74]. The gene duplication processes, including segmental, tandem, and whole-genome duplications, are the main driving forces for expanding the gene family in different plant species [75,76]. Chromosomal mapping of TaPIN genes revealed that the 44 TaPINs were not uniformly distributed on 21 chromosomes (Figure 2A,C). The gene number on each chromosome varied from one to five, with chromosomes 1A, 1B, 1D, 4A, 4B, 4D, 6A, 6B and 6D having a single gene, while chromosome 7A had three genes, chromosomes 3A, 3B, 3D, 5A, 5B, 5D and 7D contained four genes, and chromosome 7B had five genes. Gene duplication analysis showed 15 pairs of duplicated genes which shared high nucleotide sequence similarity. The duplicated pairs are TaPIN25:TaPIN28, TaPIN24:TaPIN27, TaPIN21:TaPIN29, TaPIN22:TaPIN26, TaPIN34:TaPIN42, TaPIN7:TaPIN15, TaPIN9:TaPIN13, TaPIN6:TaPIN10, TaPIN37:TaPIN38, TaPIN39:TaPIN43, TaPIN1:TaPIN2, TaPIN8:TaPIN12, TaPIN17:TaPIN18, TaPIN30:TaPIN32 and TaPIN36:TaPIN41. Further, the Ka/Ks ratios of 15 gene pairs were less than 1, revealing that TaPIN genes underwent a strong purifying selection pressure (Figure S4 and Table S2). The expansion of the TaPIN gene family might be due to the natural whole-genome duplication events. OsPIN1a-1d and three OsPIN5 are the results of segmental duplications in rice [47]. Gene duplication events were also reported in the three maize PINs, such as ZmPIN1a-1c [74]. Our gene duplication analysis revealed that the TaPIN gene duplication events were similar, as previously described in rice and maize. Therefore, these results demonstrate that whole-genome duplication and segmental duplications might play an important role in expanding and evolving the TaPIN genes.

To examine the synteny relationships of PIN genes in wheat and different plant species, we identified 31, 38, 68, 40 and 36 orthologous gene pairs between TaPINs with other PIN genes in *Ae. tauschii*, *B. distachyon, T. dicoccoides*, *O. sativa* and *A. thaliana*, respectively (Figure 3 and Table S3). In addition, *B. distachyon,* (BB, diploid) and *Ae. tauschii* (DD, diploid) were the natural foundations of the B and D subgenomes of hexaploid wheat. The synteny relationship revealed that eleven orthologous gene pairs between *Ae. tauschii* and a wheat D subgenome were located on the same chromosomes, with one on 1D, four on 3D, one on 4D, one on 5D, and four on 7D (Figure 3 and Table S3). Further, twenty-four orthologous gene pairs between *T. dicoccoides* and a wheat AABB subgenome were located on the same chromosomes, with one on 1A, four on 3A, one on 4A, two on 5A, one on 6A, two on 7A, one on 1B, four on 3B, one on 4B, two on 5B, one on 6B, and four on 7B (Figure 3 and Table S3). These results indicate that these PIN genes might be derived from *Ae. tauschii* and *T. dicoccoides* orthologous genes during hybridization events. Additionally, more orthologous gene pairs were identified between wheat and *O. sativa* and *A. thaliana*, which showed that TaPIN and other PIN genes in these plant species might have originated from a common ancestor during the evolutionary process.

The gene structure analysis of TaPINs demonstrated that the gene structure is highly conserved for most of the TaPIN genes. Twenty-three out of fourty-four TaPIN genes contain two exons (Figure 4). *TaPIN14*, *TaPIN31* and *TaPIN35* contain four exons, while *TaPIN1*, *TaPIN2*, *TaPIN3*, *TaPIN4*, *TaPIN18*, *TaPIN26*, *TaPIN36* and *TaPIN37* had three exons, and *TaPIN5*, *TaPIN7*, *TaPIN9*, *TaPIN12*, *TaPIN13*, *TaPIN22*, *TaPIN42* and *TaPIN43* had only a single exon. Intron size is a crucial factor that influences the gene size; for instance, the remarkable difference in gene size observed between the biggest gene *TaPIN35* (2.8 kb) and the smallest gene *TaPIN24* (1.1 kb) was due to the difference in the total intron length (1.1 kb vs. 0.1 kb). This conserved exon/intron organization of PIN genes was also found in other plant species [34,47,52,74,77]. In order to elucidate structural comparisons among TaPIN proteins, we identified the ten conversed motifs (Figure 5A,B). Conserved motif analysis also revealed two distinct types of motif compositions observed among the TaPIN proteins. We found that seven motifs were common in all the TaPIN proteins (Motif 1-7). Bennett et al. [43] classified the Arabidopsis PIN proteins into two groups: long or canonical PINs (AtPIN1-4 and AtPIN6-7) and short or noncanonical PINs (AtPIN5, AtPIN8). The TaPIN gene family consists of 18 members from long or canonical PINs (AtPIN1-4), including TaPIN1, TaPIN2, TaPIN3, TaPIN4, TaPIN8, TaPIN12, TaPIN16, TaPIN17, TaPIN18, TaPIN30, TaPIN31, TaPIN32, TaPIN33, TaPIN35, TaPIN36, TaPIN40, TaPIN41 and TaPIN44 (166 to 515 amino acids), while the short or noncanonical PINs comprise 26 members from groups AtPIN5 and AtPIN8 (168–256 amino acids). Further, TaPIN proteins share two highly conserved hydrophobic regions located at the N- and C-terminal and connected by a central hydrophilic loop (Figure S6). All TaPIN proteins have 8–10 transmembrane domains except for TaPIN43, which had only 5. In addition, multiple sequence alignment of TaPIN proteins also displayed conserved amino and carboxy-terminal regions of transmembrane segments with a variable central hydrophilic region (Figure S7). The length of the central hydrophilic region was approximately 350 amino acids for the long or canonical PIN members and 40–100 amino acids for the short or noncanonical PIN members. Thus, these results indicate that with the divergence of monocot and dicot plants, there had been significant changes in the number and the structure of PINs. Determination of the subcellular localization of TaPIN proteins will help understand the molecular function. Most of the TaPIN proteins were predicted to be located on the plasma membrane (Table 1). The plasma membrane-localized long PINs are involved in polar auxin transport, while ER-localized short PINs regulate intracellular auxin homeostasis [2,22,35,38]. Therefore, these results also suggest that PIN genes in wheat could have a similar function.

### 3.2. Expression Profiling of TaPIN Genes in Various Tissues and Developmental Stages Indicating Their Role in Plant Growth and Development

The cis-acting regulatory element is a non-coding DNA sequence that exists in the promoter region of genes. The distribution of different CAREs in promoter regions may reveal differences in the regulation and function of genes [78]. The identified CAREs elements in this study were classified into three main categories: phytohormone response, related to stress response, and growth and development (Figure 7). More than 10 CAREs were identified in the promoter region of each TaPIN (Table S5). A total of 19 cis-elements related to light response were detected, such as ATCT motif, Box 4, and AE-box (part of a conserved DNA module involved in light responsiveness), Box II, TCT-motif, GTGGC-motif, Gap-box, I-box, chs-CMA1a, chs-CMA2a, L-box, GATA-motif, and TCCC-motif (part of a light-responsive element), ACE and G-Box (cis-acting elements involved in light responsiveness), and Sp1, MRE, GT1-motif, and 3-AF1 binding site (light-responsive elements) [79,80]. We also predicted six cis-elements related to growth and development including CAT-box (meristem expression), RY-element (seed-specific regulation), CAAAGATATC-motif (circadian control), GCN4-motif (endosperm expression), MSA-like (cell cycle regulation), and O2-site (zein metabolism regulation) [81,82]. Further, we also examined the CARE related to hormone response in the promoter of TaPIN genes. ABRE is a cis-acting element involved in abscisic acid responsiveness, and the differential expression of PIN genes upon ABA treatment was shown in different plants [44,49,52,74]. The ABRE cis-elements were predicted in most TaPIN genes except TaPIN1, TaPIN2, TaPIN3, TaPIN5, TaPIN9, TaPIN13 and TaPIN26. Subsequently, we also found some other hormone-related cis-elements such as TCA-element (salicylic acid responsiveness), P-box and TATC-box (gibberellin-responsive element), CGTCA-motif (MeJA responsiveness), TGA-element, and AuxRR-core (auxin-responsive element) [83]. Moreover, other cis-elements have been implicated in diverse stress conditions, including MBS (drought inducibility), LTR (low-temperature responsiveness), and TC-rich repeats (defense and stress responsiveness), which were also predicted in the TaPIN promoters. Previous studies have shown that soybean PIN genes were differentially expressed upon ABA and auxin treatments [49]. The majority of the cotton PIN genes were responsive to auxin and salicylic acid in shoots and roots [44]. Similarly, all maize auxin transporter genes were also identified to be IAA-responsive and also showed different expression levels in diverse abiotic stresses [84]. Collectively, these results demonstrate that PIN gene family members in wheat might be regulated by a wide range of developmental processes, various hormones, and stress; of course, this needs to be confirmed in the future by experimental studies. These data will provide valuable insights to understand the TaPIN gene family in response to phytohormones, stress, and growth and development. Furthermore, the application of genome editing technology might lead to better understanding the function of TaPIN genes.

Several studies have demonstrated the role of PIN proteins in numerous developmental processes and in response to environmental stimuli [2,4,6,10,22,34,43]. Expression profiling of the TaPIN gene family revealed that TaPIN expression was observed in different tissues such as *TaPIN6*, *TaPIN10*, *TaPIN11*, *TaPIN14*, *TaPIN19*, *TaPIN23*, *TaPIN26*, *TaPIN29*, *TaPIN34*, *TaPIN39*, *TaPIN42* and *TaPIN43*, which were elevated in roots (Figure 8). *TaPIN4*, *TaPIN8*, *TaPIN12*, *TaPIN16*, *TaPIN17*, *TaPIN18*, *TaPIN30*, *TaPIN37* and *TaPIN39* were highly expressed in the spike. Further, *TaPIN7*, *TaPIN15*, *TaPIN19* and *TaPIN22* showed induction in the leaf. In addition, *TaPIN31*, *TaPIN32*, *TaPIN35*, *TaPIN40*, and *TaPIN44* expression was raised in the grain, while *TaPIN5*, *TaPIN9*, *TaPIN13*, *TaPIN21* and *TaPIN28* showed higher expression in the stem. The PIN proteins have been implicated in a variety of developmental processes, including leaf venation and vascular development, gravitropism, apical dominance, phototropism, embryo axis formation, and root development [4,5,6,8,10,18,26,70]. Different PIN proteins differentially catalyze auxin transport in the root hair cell of Arabidopsis [85]. AtqPIN1 and AtqSoPIN1 perform distinct functions and regulate auxin transport during bulbil formation in *Agave tequilana* [86]. Further, overexpression of the ABP1 altered PIN-mediated auxin transport in tobacco has been observed [56]. NtPIN4 regulates axillary bud growth in tobacco [51]. OsPIN1b regulates seminal root elongation in response to low phosphate and nitrogen in rice [57]. MtPIN1 and MtPIN3 regulate the shade avoidance response under various environments in *M. truncatula* [58]. Our gene ontology analysis also suggested multiple roles of TaPIN genes in the cell (Figure S8A–C). Therefore, this temporal and spatial expression pattern of TaPIN genes indicates that these PINs might have a function in different tissues and various developmental stages in wheat.

### 3.3. TaPIN Gene Family Members Respond to Biotic and Abiotic Stresses in Wheat

The PIN gene family plays a critical role in a plant’s adaptations to internal and external stimuli at both transcriptional and post-transcriptional levels. Our results also show that three TaPIN genes (*TaPIN5*, *TaPIN6* and *TaPIN10*) were highly induced upon Septoria infection (Figure S10), while the expression of seven TaPIN genes (*TaPIN13*, *TaPIN18, TaPIN19*, *TaPIN20*, *TaPIN25*, *TaPIN35* and *TaPIN44*) was significantly elevated during powdery mildew infection. Most of the TaPINs respond similarly to powdery mildew; this is why PM expression profiles cluster together (Figure S9B). Further, five TaPIN genes (*TaPIN7, TaPIN11, TaPIN15, TaPIN22* and *TaPIN26*) responded to drought stress, only two TaPIN genes (*TaPIN14* and *TaPIN37*) were induced during heat stress, and three TaPINs (*TaPIN8, TaPIN9* and *TaPIN28*) responded to the combined heat and drought stress (Figure S10). Several PIN genes in sorghum, maize, and soybean were found to be responsive to different abiotic stresses such as drought and salt [49,52,74]. PIN genes may be commonly used in different plant species to adapt to various stress conditions. AtPIN2 is also required to maintain root growth under alkaline stress by altering the proton secretion in Arabidopsis [87]. Another study also showed that modulation in the intracellular trafficking of AtPIN2 and AtPIN3 was responsible for the impeded polar auxin transport in cold stress [88]. Moreover, expression profiling of TaPINs was further validated by qRT-PCR. The qRT-PCR results also exhibit a similar expression with slight variation (Figure 9). The expression pattern of TaPIN genes under biotic and abiotic stress revealed that they might be involved in the stress tolerance in wheat. Thus, these results demonstrate that wheat responds to diverse stresses through an intricate gene network, which requires coordinated regulation among the TaPINs.

Several studies have demonstrated that TaPIN interacts with other proteins to regulate diverse developmental processes, and hormonal and stress responses. P-glycoproteins 1 and 19 interact with PIN1, and PIN2 enhances auxin transport activity in Arabidopsis [13]. The serine/threonine-protein kinase PINOID (PID) catalyzes PIN phosphorylation, regulating the apical and basal PIN polarity in Arabidopsis [89]. Our STRING database predicted results also reveal that TaPIN32 might interact with TaPIN8 and TaPIN44 (Figure 10 and Table S8); however, TaPIN32 was co-expressed with TaPIN8 and TaPIN44 in the spike, in grain development, and under biotic stress (Figure 8 and Figure S10), suggesting that TaPIN8, TaPIN32 and TaPIN44 might perform essential functions in the spike and grain development and during powdery mildew infection through interacting with each other. The expression of TaPIN8 and TaPIN9 was significantly elevated in the combined drought and heat stress in 1 h, potentially interacting with Traes_5DS_2B0D2D352.1 (serine/threonine-protein kinase: PINOID), and PINOID specifically phosphorylates the PIN proteins, suggesting that it might regulate the localization of the wheat PIN protein and polar auxin transport through changes in the phosphorylation status during abiotic stress conditions (Figure S10). TaPIN genes also interacted with the ABC transporter and other auxin efflux carrier proteins. These results provide valuable information to further elucidate the precise biological functions of TaPIN genes to develop the high-yielding and stress-tolerant varieties in wheat.

Moreover, based on the expression profiling of TaPIN genes, we proposed a possible working model in Figure S11 to show the functions of the TaPIN genes in a variety of biological processes in wheat. 

This model demonstrates that endogenous and external stimuli stimulated the expression of TaPIN genes. These signals were sensed by the different cis-regulatory elements and regulate the expression and functions of TaPIN genes implicated in various plant developmental processes and stress conditions, which eventually affect the growth and tolerance mechanism against diverse stress conditions. In summary, our work provides valuable information about the TaPIN gene family, TaPINs’ functions in the various plant developmental processes, and their response to hormones and stress. Therefore, this study provides putative candidate genes for improving plant growth and stress tolerance and also facilitates a better understanding of the various developmental processes in common wheat.

## 4. Materials and Methods

### 4.1. Identification of PIN Genes in the Wheat Genome

To perform genome-wide analysis in bread wheat, genome data (IWGSC) were retrieved from the Ensembl Plants website (http://plants.ensembl.org/index.html accessed on 10 May 2021). Two methods were used to identify putative PIN genes in wheat. In the first approach, we made a local database of the protein sequences of bread wheat in BioEdit v7.2.6 [90]. The twenty PIN genes from Arabidopsis and rice were used to identify putative PIN genes in bread wheat in the local database using BLASTp. The e-value of 10^−5^ and > 100-bit scores were kept cut-off to identify putative PIN genes, and, finally, the BLASTp output was tabulated. In the second approach, the protein sequences of PINs from other plant species were downloaded from Ensembl hosts the latest wheat assembly from the IWGSC (RefSeq v1.0) (http://plants.ensembl.org/index.html accessed on 10 May 2021), and a BLASTp search was executed against the *T. aestivum* proteome with bit-score > 100 and an e-value cut-off of 10^−5^. Based on both above methods, putative PIN candidates were selected. After removing redundant results, the remaining sequences were further verified for the existence of transmembrane domains using other databases: Simple Modular 132 Architecture Research Tool tool (SMART, http://smart.emblheidelberg.de/ accessed on 10 May 2021), InterPro (https://www.ebi.ac.uk/interpro) NCBI CDD (https://www.ncbi.nlm.nih.gov/Structure/cdd/cdd.shtml accessed on 10 May 2021), and HMMscan (https://www.ebi.ac.uk/Tools/hmmer/search/hmmscan accessed on 10 May 2021), and the sequences lacking transmembrane domains were removed. Finally, the protein sequences with PIN-related domains were taken and named sequentially according to their locations on the chromosomes.

### 4.2. Chromosome Localization and Gene Duplication

For the distribution on chromosomes, genomic positions of PIN genes were downloaded from the Ensembl Plants BioMart (http://plants.ensembl.org/biomart/martview accessed on 11 May 2021). The PIN genes were named with a ‘Ta’ prefix and numbered in ascending order with their increasing position on the chromosome. PhenoGram (http://visualization.ritchielab.org/phenograms/plot accessed on 11 May 2021) was used to represent TaPIN genes on the wheat chromosomes. MCScanX tool kit was used to investigate gene duplication events within species and similarity between PIN genes in wheat and other plant species [91]. The non-synonymous substitution rate (Ka), synonymous substitution rate (Ks), and the Ka/Ks ratio were calculated using TBtools [92].

### 4.3. Physico-Chemical Characteristics, Subcellular Localization, the Transmembrane Domain, and 3D Structure

The protein characteristics, including the isoelectric point, lengths, and molecular weight of TaPIN proteins, were evaluated using the isoelectric point calculator [93,94] and ExPASy (https://web.expasy.org/compute_pi/ accessed on 12 May 2021). Subcellular localization was predicted using CELLO (http://cello.life.nctu.edu.tw/ accessed on 12 May 2021). The transmembrane helix and topology of the TaPINs were analyzed using SOSUI (http://www.cbs.dtu.dk, http://harrier.nagahama-i-bio.ac.jp accessed on 12 May 2021), TMHMM (http://www.cbs.dtu.dk/services/TMHMM/ accessed on 12 May 2021), and MEMSAT-SVM available in the Phyre2 server. The three-dimensional (3D) structure of TaPINs was produced using the Phyre2 server (http://www.sbg.bio.ic.ac.uk/phyre2/html/page.cgi?id=index).

### 4.4. Gene Structure, Gene Ontology, and Motif Analysis

The CDS and genomic and protein sequences of wheat PIN genes were retrieved from the Ensembl Plants BioMart (http://plants.ensembl.org/biomart/martview). Intron and exon positions and untranslated regions were visualized using the Gene Structure Display Server 2.0 (http://gsds.gao-lab.org/). For gene ontology, TaPIN protein sequences were used to predict gene ontology terms using agriGO [95] and EggNOG (http://eggnogdb.embl.de/#/app/emapper). The conserved motifs in the TaPINs were elucidated using the MEME tool (http://meme-suite.org/tools/meme) with default settings.

### 4.5. Cis-acting Regulatory Elements (CAREs) Analysis and Protein Interaction Network

To identify CAREs, 2000 bp upstream sequences of PIN genes were downloaded from Ensembl Plants and analyzed using the PlantCARE online server (http://bioinformatics.psb.ugent.be/webtools/plantcare/html/ accessed on 15 May 2021). The number of occurrences for each CARE motif was counted for TaPIN genes, and the most commonly occurring CAREs were used to produce Figure 7 in TBtools [92]. The TaPIN protein interaction network was examined using the STRING online server (https://string-db.org/cgi accessed on 15 May 2021).

### 4.6. Expression Profiling of TaPIN Genes

Transcripts per million (TPM) values for five tissues (leaf, stem, root, spike and grain) and under diverse stress conditions were retrieved from Wheat Expression Browser (http://www.wheat-expression.com/ accessed on 16 May 2021). Heatmaps and principal component analysis (PCA) plots were produced using clustvis (https://biit.cs.ut.ee/clustvis/) and TBtools software [92].

### 4.7. Plant Material, Growth Conditions, Drought, and Heat Treatment

Seeds of wheat were sown on soil in plastic pots and reared in a greenhouse. Ten-day-old wheat seedlings were acclimatized for two days in growth chamber conditions. They were further subjected to drought and high-temperature stress (37 °C) for 1 h and 6 h. Controls were kept at 25 °C. The drought- and high temperature-stressed seedlings were collected for RNA extraction and stored at −80 °C.

### 4.8. RNA Isolation and Real-Time PCR

RNA was isolated from control, drought-, and heat-treated plants as described by [96,97]. To remove the genomic DNA contamination from RNA, samples were treated with *DNase*-I (Takara Bio. Inc., Shiga, Japan) at 37 °C for 30 min. cDNA was prepared using the iScriptTM cDNA synthesis kit (Bio-Rad, Hercules, CA, USA) at 46 °C for 20 min. cDNA was quantified using a NanoDrop (ND-1000, NanoDrop Technologies, Wilmington, DE, United States). Quantitative real-time PCR (qRT-PCR) was performed using the Applied Biosystems 7500 Fast Real-Time PCR (Applied Biosystems, Massachusetts, United States). Each qRT-PCR reaction was carried out with three technical replicates and repeated three times. The fold change was calculated based on mean 2^−*ΔΔCT*^ values [98]. Finally, this value was used for plotting graphs. Wheat actin (AB181991) was used as the internal control to normalize the data. Primer pairs were designed using PrimerQuest Tool (https://sg.idtdna.com/PrimerQuest/Home/Index accessed on 17 May 2021), and primers used in this work are listed in Table S9. 

## 5. Conclusions

Bread wheat is an important cereal crop and staple food worldwide. Thus, all plant scientists have been concentrated on increasing yield, and on improving the quality and stress tolerance in wheat. The PIN gene family is involved in plant growth and development. In the present study, we identified and characterized the PIN gene family in wheat. Expression profiling showed the role of TaPINs in various developmental stages and stress conditions. The outcome of this study will be helpful to understand the role of PINs in plant developmental processes and diverse stress conditions and their sequential execution to increase yield and develop stress-tolerant varieties of wheat.

## Figures and Tables

**Table 1:** Nomenclature and characteristics of the putative PIN-FORMED (PIN) proteins in wheat.

Proposed Gene Name	Gene ID	Genomic Location	Orientation	CDS Length (bp)	Intron Number	Protein Length (aa)	Molecular Weight (kDa)	Isoelectric Point (pI)	GRAVY	Predicted Subcellular Localization
TaPIN1	TraesCS1A02G415400	1A:574359017–574359996	Reverse	1800	2	599	63.57	8.632	0.354	Plasma membrane
TaPIN2	TraesCS1B02G445400	1B:665407466–665408862	Reverse	1794	2	597	63.37	8.989	0.297	Plasma membrane
TaPIN3	TraesCS1D02G422900	1D:478154308–478155466	Reverse	1791	2	596	63.18	9.12	0.321	Plasma membrane
TaPIN4	TraesCS3A02G231500	3A:432041807–432043538	Forward	1788	2	595	63.72	6.619	0.325	Plasma membrane
TaPIN5	TraesCS3A02G243700	3A:456920851–456922269	Reverse	1074	0	357	39.19	8.603	0.795	Plasma membrane
TaPIN6	TraesCS3A02G331300	3A:575870221–575871321	Reverse	1299	1	432	45.98	8.819	0.554	Plasma membrane
TaPIN7	TraesCS3A02G426700	3A:669623930–669624178	Reverse	1134	0	377	40.55	9.161	0.699	Plasma membrane
TaPIN8	TraesCS3B02G260700	3B:418660347–418660613	Forward	1830	1	609	65.38	7.54	0.243	Plasma membrane
TaPIN9	TraesCS3B02G276500	3B:446634435–446635854	Forward	1074	0	357	39.16	8.603	0.809	Plasma membrane
TaPIN10	TraesCS3B02G361500	3B:572835666–572837001	Reverse	1287	1	428	45.74	9.064	0.537	Plasma membrane
TaPIN11	TraesCS3B02G462900	3B:705706664–705706909	Reverse	1107	1	368	39.7	8.998	0.675	Plasma membrane
TaPIN12	TraesCS3D02G221900	3D:302847280–302848826	Reverse	1773	0	590	62.69	6.465	0.319	Plasma membrane
TaPIN13	TraesCS3D02G247700	3D:346727914–346729331	Forward	1074	0	357	39.1	8.603	0.809	Plasma membrane
TaPIN14	TraesCS3D02G324800	3D:437732979–437734144	Reverse	1296	3	431	45.92	8.689	0.592	Plasma membrane
TaPIN15	TraesCS3D02G421600	3D:533875425–533875673	Reverse	1137	1	378	40.49	9.147	0.714	Plasma membrane
TaPIN16	TraesCS4A02G188100	4A:466939085–466940349	Reverse	1707	1	568	60.78	8.361	0.336	Plasma membrane
TaPIN17	TraesCS4B02G130100	4B:170928310–170929402	Forward	1701	1	566	60.55	8.374	0.328	Plasma membrane
TaPIN18	TraesCS4D02G125300	4D:109470858–109471950	Forward	1701	2	566	60.59	8.361	0.327	Plasma membrane
TaPIN19	TraesCS5A02G284500	5A:492633348–492636528	Reverse	1098	0	365	38.67	8.28	0.714	Plasma membrane
TaPIN20	TraesCS5A02G285700	5A:493780035–493782506	Forward	1164	0	387	41.36	8.86	0.559	Plasma membrane
TaPIN21	TraesCS5A02G286000	5A:493815389–493817262	Reverse	1074	0	357	37.92	8.948	0.606	Plasma membrane
TaPIN22	TraesCS5B02G283500	5B:469121420–469124421	Reverse	1107	0	368	38.84	8.28	0.699	Plasma membrane
TaPIN23	TraesCS5B02G283600	5B:469292031–469299978	Reverse	1095	1	364	38.96	7.519	0.621	Plasma membrane
TaPIN24	TraesCS5B02G284900	5B:470436673–470438746	Forward	1134	1	377	40.51	8.149	0.606	Plasma membrane
TaPIN25	TraesCS5B02G285000	5B:470512790–470515294	Forward	1164	1	387	41.47	9.017	0.517	Plasma membrane
TaPIN26	TraesCS5D02G291800	5D:389457955–389460969	Reverse	1104	2	367	38.87	8.868	0.672	Plasma membrane
TaPIN27	TraesCS5D02G293000	5D:390372139–390374171	Forward	1134	1	377	40.4	8.147	0.621	Plasma membrane
TaPIN28	TraesCS5D02G293100	5D:390418535–390421740	Forward	1164	1	387	41.48	8.838	0.537	Plasma membrane
TaPIN29	TraesCS5D02G293300	5D:390504794–390506554	Forward	1134	1	377	40.03	9.059	0.634	Plasma membrane
TaPIN30	TraesCS6A02G308600	6A:543394829–543395365	Forward	1767	1	588	63.76	8.236	0.174	Plasma membrane
TaPIN31	TraesCS6B02G337300	6B:593711958–593712494	Forward	1566	3	521	56.79	7.407	0.011	Plasma membrane
TaPIN32	TraesCS6D02G287800	6D:397082369–397082905	Forward	1770	1	589	63.86	8.236	0.164	Plasma membrane
TaPIN33	TraesCS7A02G190600	7A:148415130–148415675	Forward	1764	1	587	64.02	8.652	0.176	Plasma membrane
TaPIN34	TraesCS7A02G258800	7A:250325040–250326673	Reverse	1104	1	367	39.23	8.126	0.654	Plasma membrane
TaPIN35	TraesCS7A02G492400	7A:681011797–681013386	Reverse	1899	1	632	67.3	9.256	0.254	Plasma membrane
TaPIN36	TraesCS7B02G095500	7B:109702420–109702965	Forward	1761	2	586	63.9	8.652	0.183	Plasma membrane
TaPIN37	TraesCS7B02G331500	7B:586716404–586721044	Forward	1221	2	406	43.87	6.619	0.536	Plasma membrane
TaPIN38	TraesCS7B02G359200	7B:621295197–621300495	Forward	1182	1	393	41.63	5.929	0.478	Plasma membrane
TaPIN39	TraesCS7B02G359300	7B:621396217–621403628	Reverse	1200	1	399	42.83	7.983	0.589	Plasma membrane
TaPIN40	TraesCS7B02G398100	7B:664108583–664110165	Forward	1899	1	632	67.26	9.256	0.252	Plasma membrane
TaPIN41	TraesCS7D02G191600	7D:146862944–146863489	Forward	1761	1	586	63.86	8.652	0.183	Plasma membrane
TaPIN42	TraesCS7D02G259700	7D:235396162–235396730	Reverse	1143	0	380	40.5	8.133	0.669	Plasma membrane
TaPIN43	TraesCS7D02G446900	7D:567514947–567515186	Reverse	858	0	285	30.22	6.456	0.496	Plasma membrane
TaPIN44	TraesCS7D02G478800	7D:589514264–589515825	Forward	1902	1	633	67.41	9.323	0.248	Plasma membrane

ID: identity; bp: base pair; aa: amino acids; pI: isoelectric point; MW: molecular weight; kDa: kilodalton.

**Table 2:** Number of PIN proteins in different plant species.

Plant Species	Genome Size (Approx.)	Coding Genes	PIN Genes
*Triticum aestivum* (6n)	17 Gb	107,891	44
*Oryza sativa* (2n)	500 Mb	37,960	12
*Arabidopsis thaliana* (2n)	135 MB	27,655	8
*Zea mays* (2n)	2.4 Gb	39,591	14
*Glycine max* (2n)	1.15 Gb	55,897	23
*Nicotiana tabacum* (4n)	4.5 Gb	61,526	29

## Data Availability

Not applicable.

## Supplementary Materials

The following are available online at https://www.mdpi.com/article/10.3390/ijms22147396/s1, Figure S1. Molecular weight (kDa) vs. isoelectric point plots of TaPIN genes, Figure S2. Distribution of TaPINs in the different groups of the phylogenetic tree, Figure S3. Phylogenetic analysis of TaPIN genes. A phylogenetic tree was constructed using MEGAX with the neighbor joining (NJ) method and 1000 bootstrap replications. Black asterisk indicates the duplicated genes, Figure S4. Chromosomal distribution and duplicated PIN gene pairs in wheat. Duplicated PIN gene pairs are connected by lines with distinct colors. The figure was generated using TBtools, Figure S5. Distribution of the number of introns of TaPINs genes in different groups of the phylogenetic tree, Figure S6. The predicted transmembrane helices of the TaPIN proteins. The transmembrane domains were identified using TMHMM2 (www.cbs.dtu.dk/services/TMHMM/) and SOSUI software tools (http://www.cbs.dtu.dk, http://harrier.nagahama-i-bio.ac.jp). The red peaks represent the predicted transmembrane domain, and blue color line indicates the central hydrophobic loop of proteins, Figure S7. Alignment of the TaPINs protein sequences. The highly conserved regions of the TaPIN proteins are underlined with black color. Colored and shaded amino acids are chemically similar residues. Dashes indicate gaps introduced to maximize the alignment of homologous region, Figure S8. Gene ontology term distribution of TaPIN gene family predicted using AgriGO. A. Biological process. B. Cellular component. C. Molecular function, Figure S9. PCA plots displaying grouping of different (A) developmental stages (B) and biotic and abiotic stress conditions based on the TaPIN expression pattern, Figure S10. Heatmap representing the expression pattern of TaPIN genes in different stress conditions. TPM values were directly used to construct the heatmap. DS: drought stress, HS: heat stress, h: hour, d: days. PM: powdery mildew, Figure S11. A possible function of TaPIN gene family in various plant developmental processes and diverse stress conditions. ABA: abscisic acid; MeJA: methyl jasmonate; SA: salicylic acid; JA: jasmonic acid; MAP: mitogen-activated protein kinase; ARF: auxin response factors; MYB: myeloblastosis; NAC: no apical meristem; ZF: zinc finger; HSF: heat shock factors, Table S1. PIN proteins from Arabidopsis, rice, and wheat used to generate phylogenetic tree, Table S2. Ratio of Ka/Ks and distribution of duplicate wheat PIN genes, Table S3. Orthologous relationships of *TaPIN* genes with other PIN genes in *B. distachyon*, *Ae. tauschii*, *T. dicoccoides*, *O. sativa* and *A. thaliana*, Table S4. Domain organization of TaPIN genes predicted using pfam with default parameters, Table S5. Cis-regulatory elements present in the *TaPIN* gene promoter region, Table S6. Significant GO terms predicted by AgriGo analysis, Table S7. Gene annotation using eggNOGmapper, Table S8. The protein–protein interaction network between TaPIN and other proteins in wheat, Table S9. qRT-PCR primers of TaPIN genes.
