# Supplementary material for: Genome-Wide Identification and Characterization of PIN-FORMED (PIN) Gene Family Reveals Role in Developmental and Various Stress Conditions in Triticum aestivum L
Source: Int J Mol Sci. 2021 Jul 9;22(14):7396. doi: 10.3390/ijms22147396 (PMC8303626; doi:10.3390/ijms22147396)
Supplement: Supplementary file 1 [file ijms-22-07396-s001.zip › TaPIN supplmentary figures.pdf]

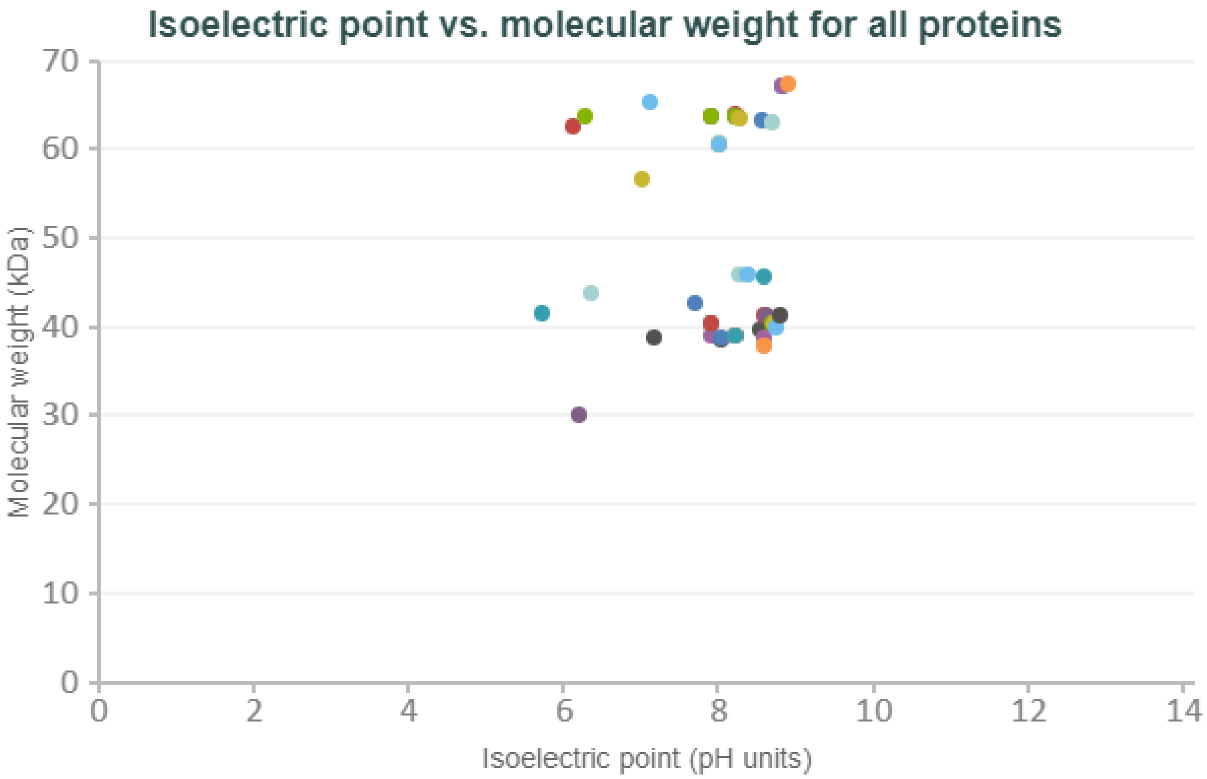

Figure S1. Molecular weight (kDa) vs. isoelectric point plots of TaPIN genes.

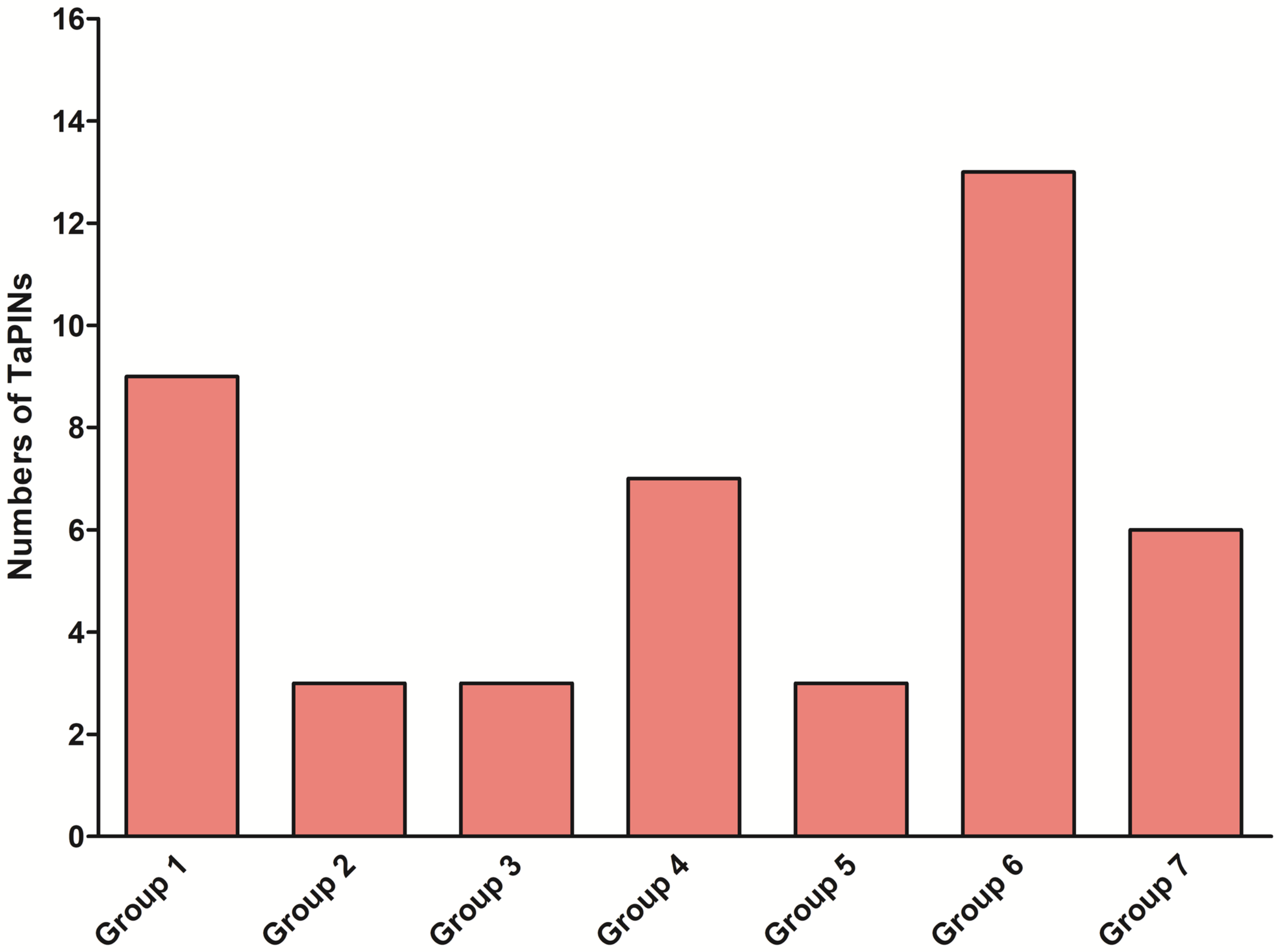

Figure S2. Distribution of TaPINs in different group of phylogenetic tree.

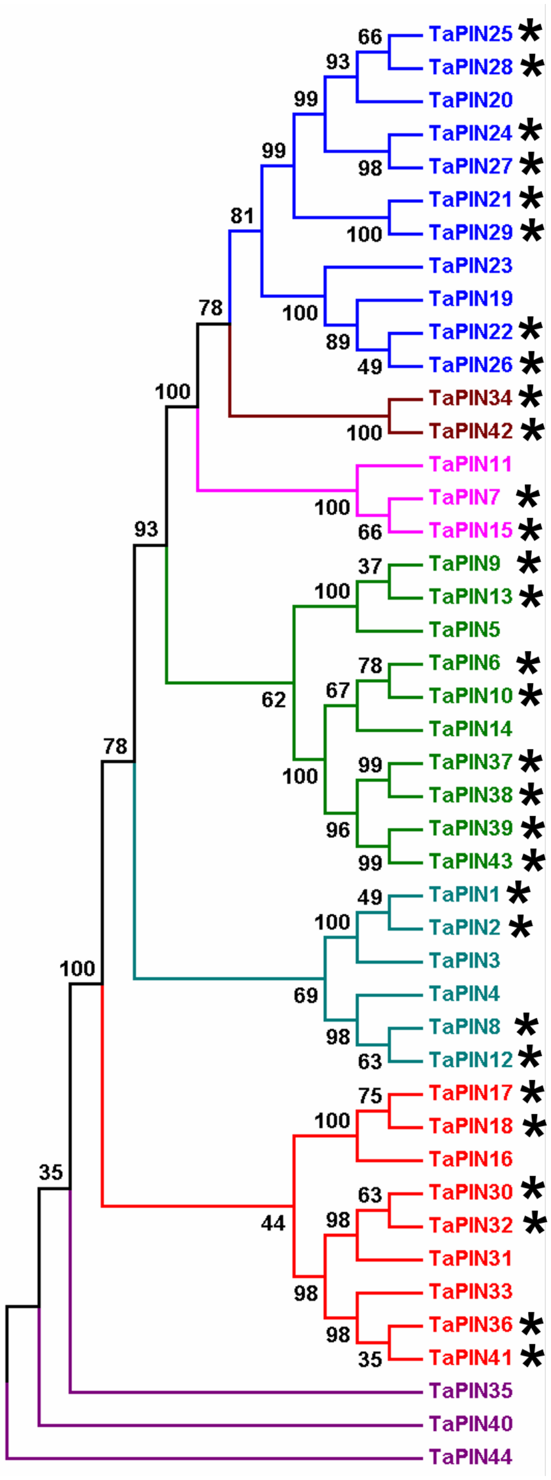
TaPIN25
TaPIN28
TaPIN20
TaPIN24
TaPIN27
TaPIN21
TaPIN29
TaPIN23
TaPIN19
TaPIN22
TaPIN26
TaPIN34
TaPIN42
TaPIN11
TaPIN7
TaPIN15
TaPIN9
TaPIN13
TaPIN5
TaPIN6
TaPIN10
TaPIN14
TaPIN37
TaPIN38
TaPIN39
TaPIN43
TaPIN1
TaPIN2
TaPIN3
TaPIN4
TaPIN8
TaPIN12
TaPIN17
TaPIN18
TaPIN16
TaPIN30
TaPIN32
TaPIN31
TaPIN33
TaPIN36
TaPIN41
TaPIN35
TaPIN40
TaPIN44

Figure S3: Phylogenetic analysis of TaPIN genes. A phylogenetic tree was constructed using MEGAX with the neighbor-joining (NJ) method and 1000 bootstrap replications. Black asterisk indicate the duplicated genes.

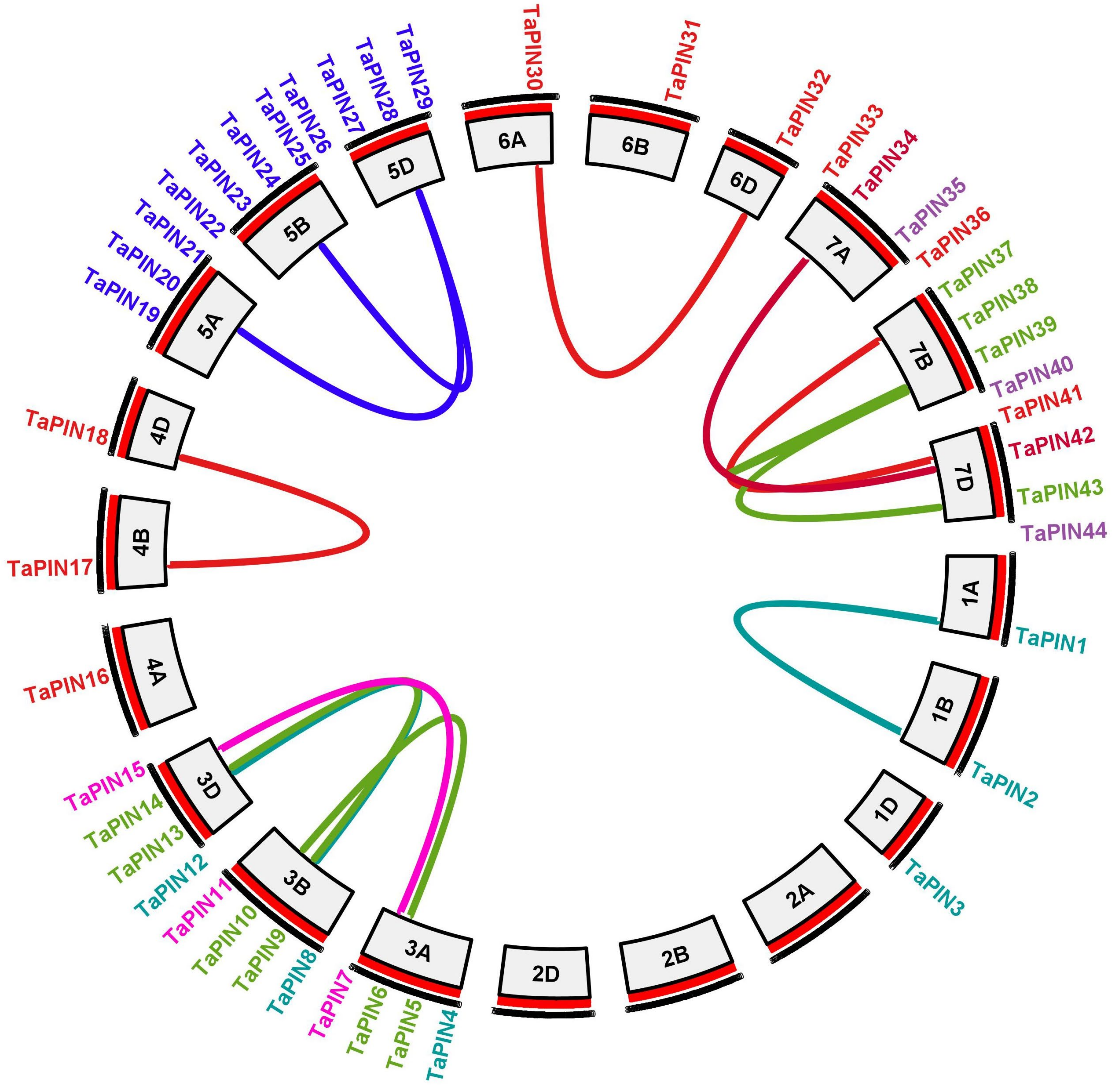

Figure S4: Chromosomal distribution and duplicated PIN gene pairs in wheat. Duplicated PIN gene pairs are connected with lines with distinct colors. The figure was generated using TBtools.

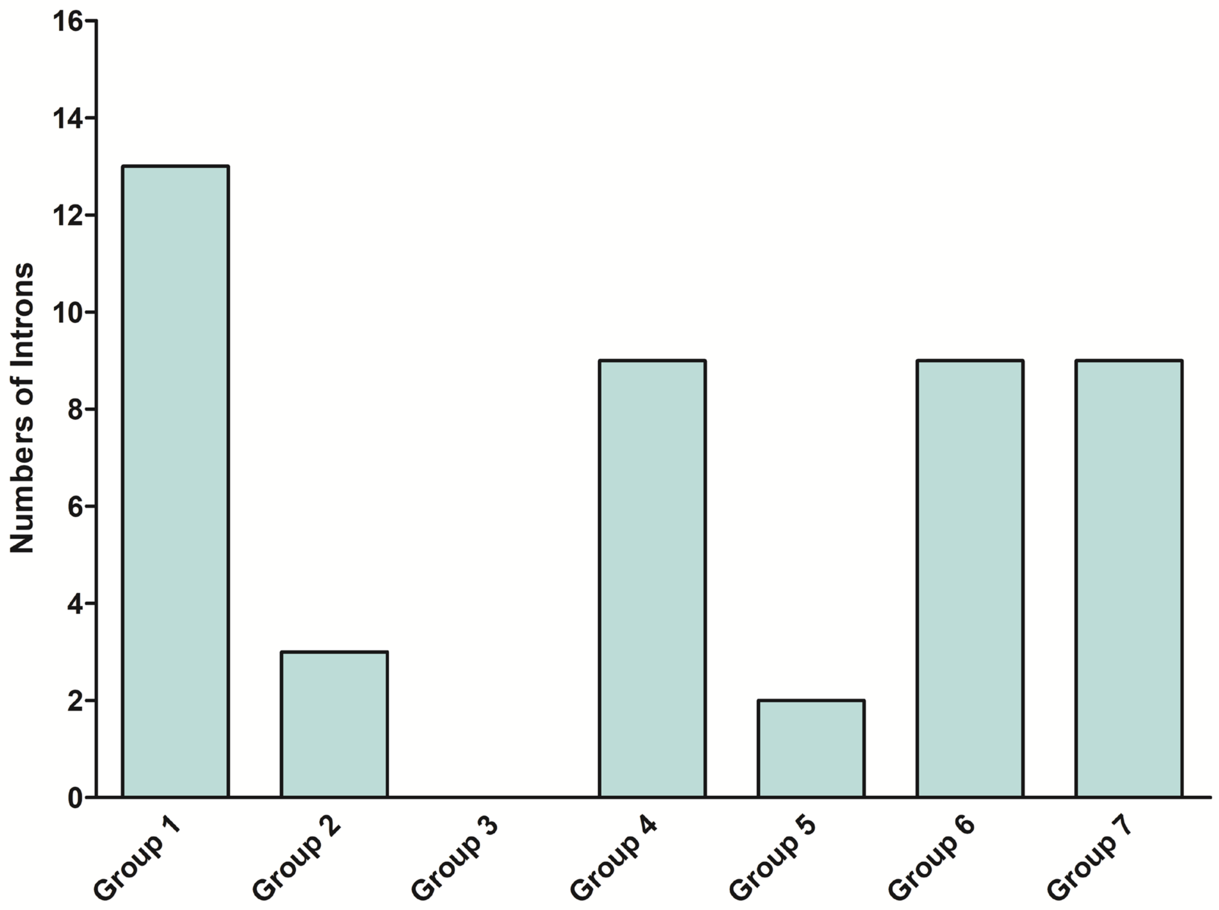

Figure S5. Distribution of number of introns of TaPINs genes in different group of phylogenetic tree.

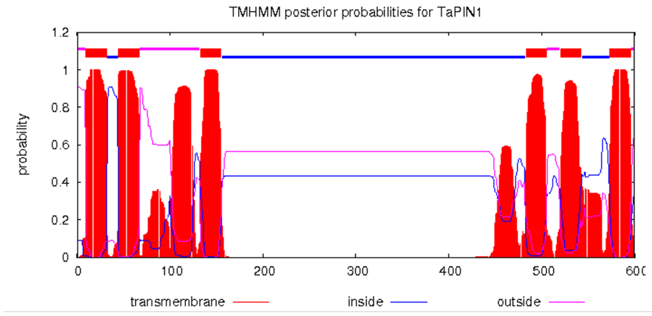
TMHMM posterior probabilities for TaPIN1
probability
transmembrane
inside
outside

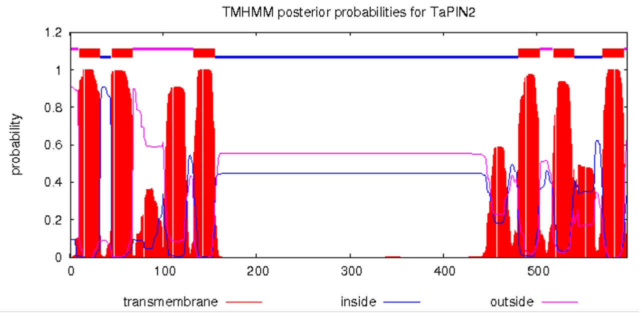
TMHMM posterior probabilities for TaPIN2
probability
transmembrane
inside
outside

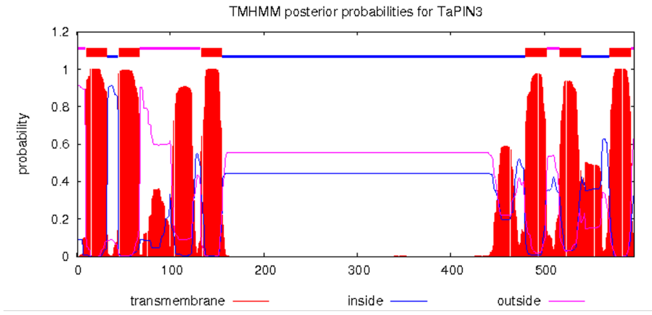
TMHMM posterior probabilities for TaPIN3
probability
transmembrane
inside
outside

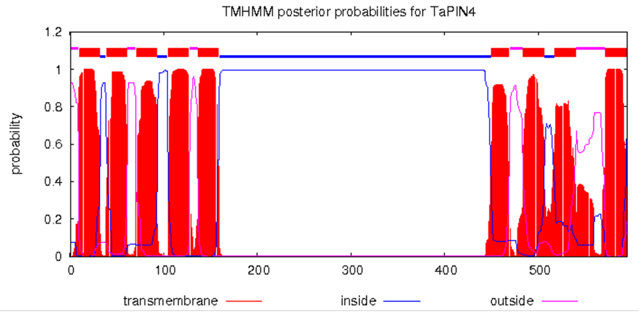
TMHMM posterior probabilities for TaPIN4
probability
transmembrane
inside
outside

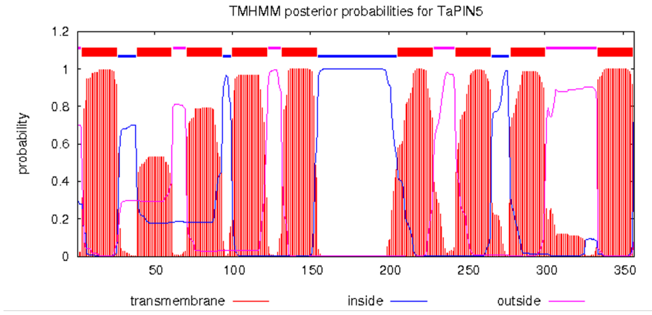
TMHMM posterior probabilities for TaPIN5
probability
transmembrane
inside
outside

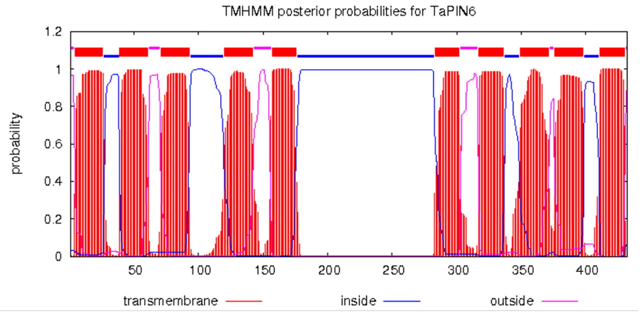
TMHMM posterior probabilities for TaPIN6
probability
transmembrane
inside
outside

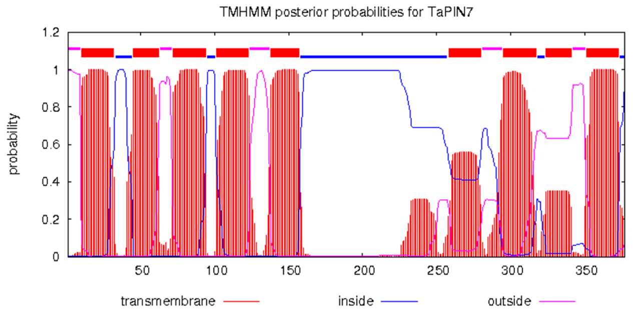
TMHMM posterior probabilities for TaPIN7
probability
transmembrane
inside
outside

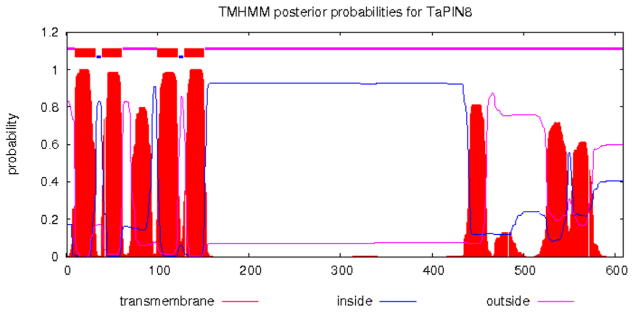
TMHMM posterior probabilities for TaPIN8
probability
transmembrane
inside
outside

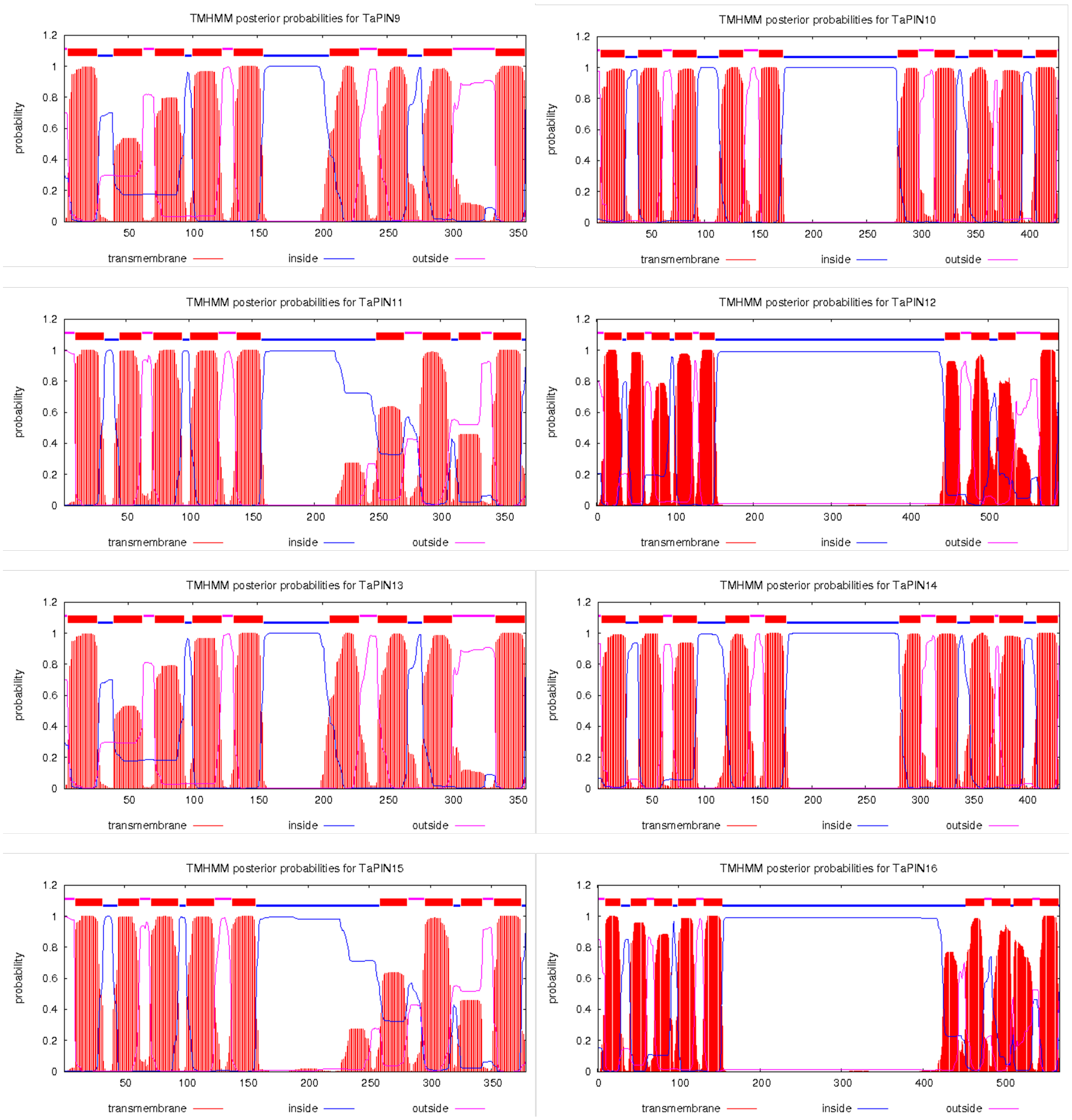
TMHMM posterior probabilities for TaPIN9
TMHMM posterior probabilities for TaPIN10
TMHMM posterior probabilities for TaPIN11
TMHMM posterior probabilities for TaPIN12
TMHMM posterior probabilities for TaPIN13
TMHMM posterior probabilities for TaPIN14
TMHMM posterior probabilities for TaPIN15
TMHMM posterior probabilities for TaPIN16
probability
transmembrane
inside
outside

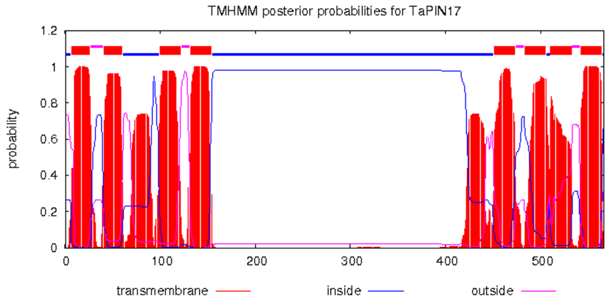
TMHMM posterior probabilities for TaPIN17
probability
transmembrane
inside
outside

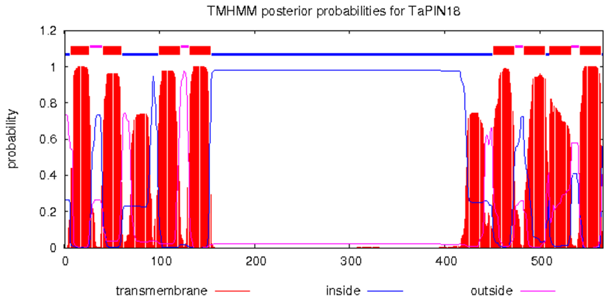
TMHMM posterior probabilities for TaPIN18
probability
transmembrane
inside
outside

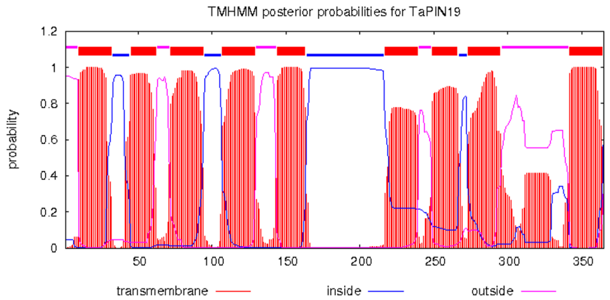
TMHMM posterior probabilities for TaPIN19
probability
transmembrane
inside
outside

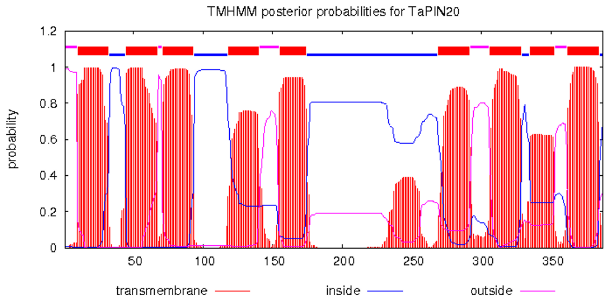
TMHMM posterior probabilities for TaPIN20
probability
transmembrane
inside
outside

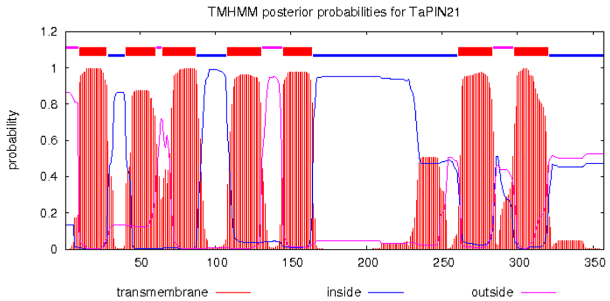
TMHMM posterior probabilities for TaPIN21
probability
transmembrane
inside
outside

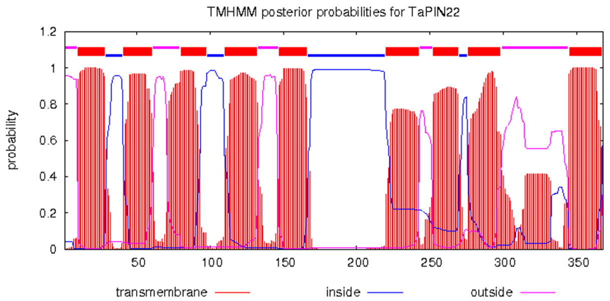
TMHMM posterior probabilities for TaPIN22
probability
transmembrane
inside
outside

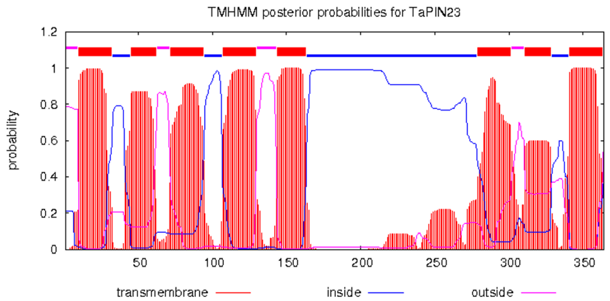
TMHMM posterior probabilities for TaPIN23
probability
transmembrane
inside
outside

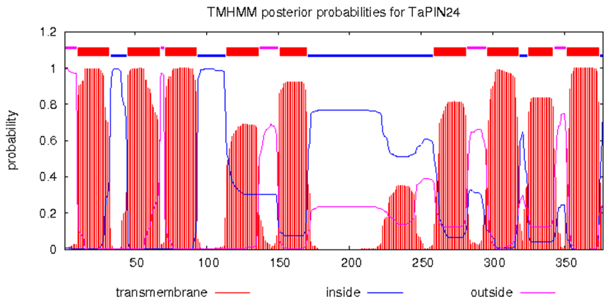
TMHMM posterior probabilities for TaPIN24
probability
transmembrane
inside
outside

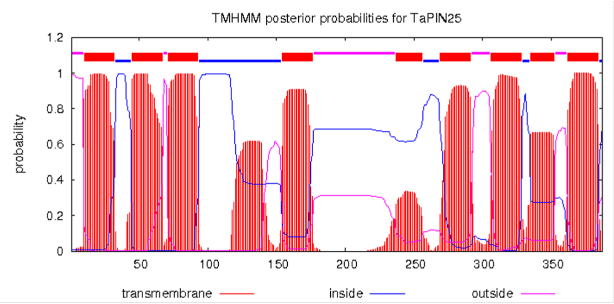
TMHMM posterior probabilities for TaPIN25
probability
0 0.2 0.4 0.6 0.8 1 1.2
50 100 150 200 250 300 350
transmembrane
inside
outside
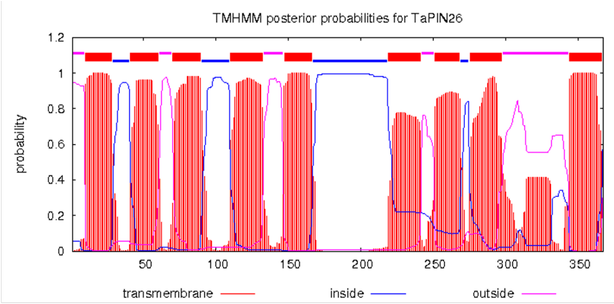
TMHMM posterior probabilities for TaPIN26
probability
0 0.2 0.4 0.6 0.8 1 1.2
50 100 150 200 250 300 350
transmembrane
inside
outside
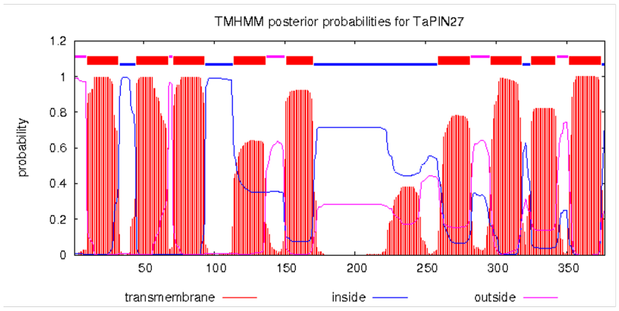
TMHMM posterior probabilities for TaPIN27
probability
0 0.2 0.4 0.6 0.8 1 1.2
50 100 150 200 250 300 350
transmembrane
inside
outside
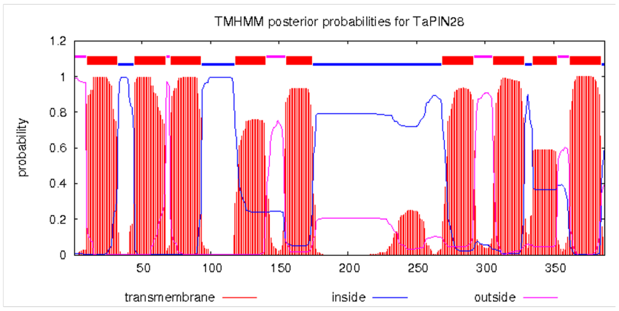
TMHMM posterior probabilities for TaPIN28
probability
0 0.2 0.4 0.6 0.8 1 1.2
50 100 150 200 250 300 350
transmembrane
inside
outside
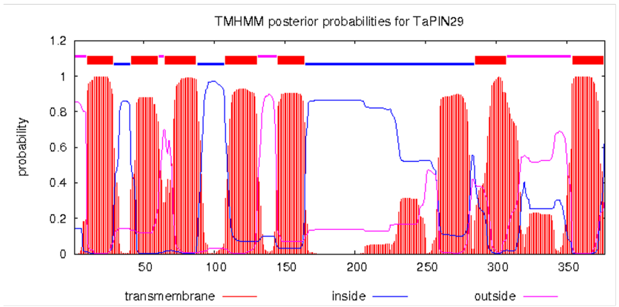
TMHMM posterior probabilities for TaPIN29
probability
0 0.2 0.4 0.6 0.8 1 1.2
50 100 150 200 250 300 350
transmembrane
inside
outside
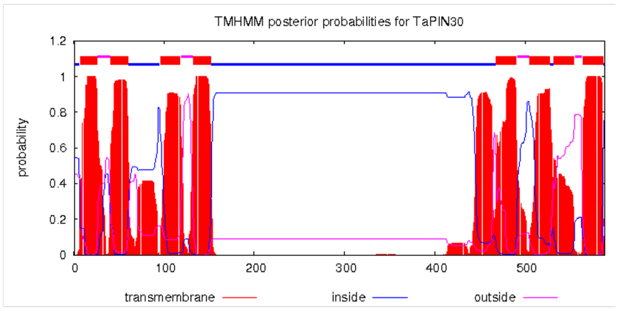
TMHMM posterior probabilities for TaPIN30
probability
0 0.2 0.4 0.6 0.8 1 1.2
0 100 200 300 400 500
transmembrane
inside
outside
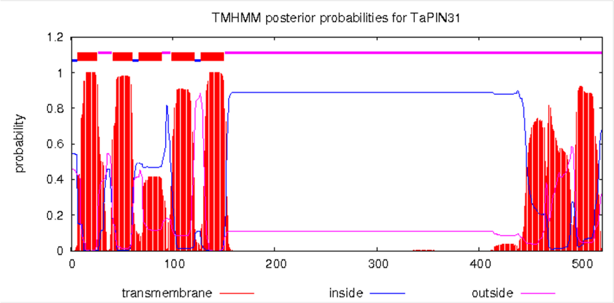
TMHMM posterior probabilities for TaPIN31
probability
0 0.2 0.4 0.6 0.8 1 1.2
0 100 200 300 400 500
transmembrane
inside
outside
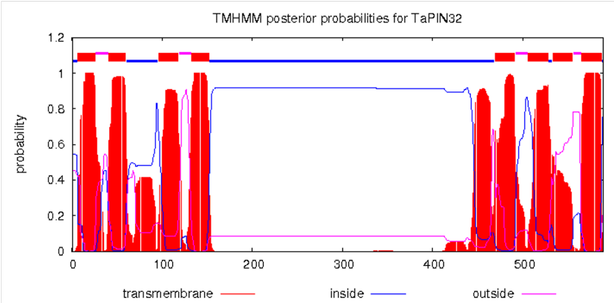
TMHMM posterior probabilities for TaPIN32
probability
0 0.2 0.4 0.6 0.8 1 1.2
0 100 200 300 400 500
transmembrane
inside
outside

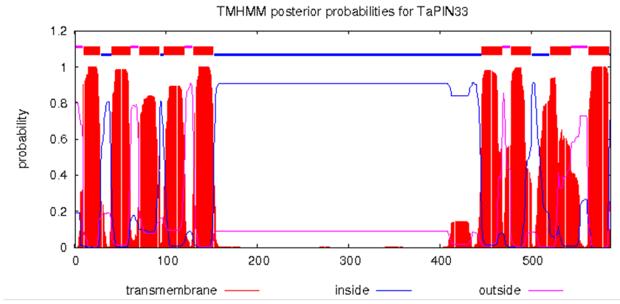
TMHMM posterior probabilities for TaPIN33
probability
transmembrane
inside
outside

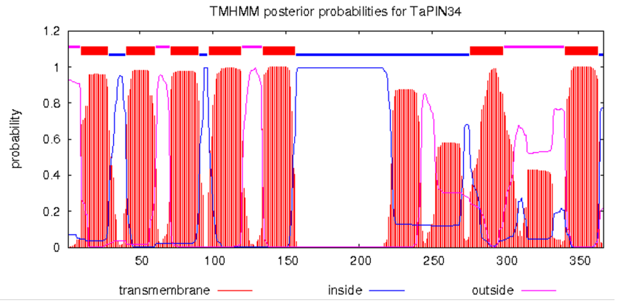
TMHMM posterior probabilities for TaPIN34
probability
transmembrane
inside
outside

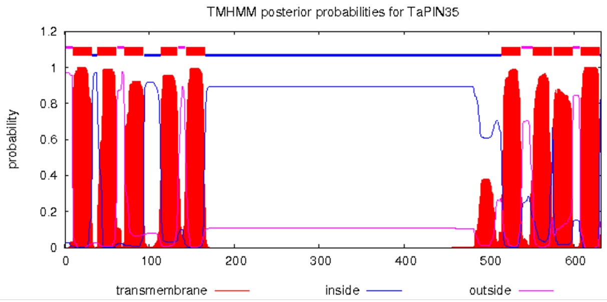
TMHMM posterior probabilities for TaPIN35
probability
transmembrane
inside
outside

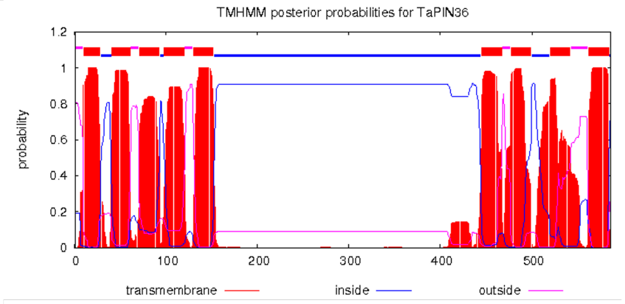
TMHMM posterior probabilities for TaPIN36
probability
transmembrane
inside
outside

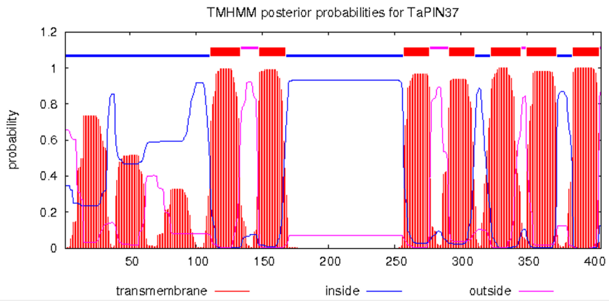
TMHMM posterior probabilities for TaPIN37
probability
transmembrane
inside
outside

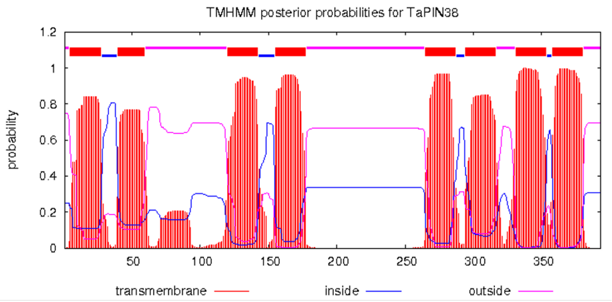
TMHMM posterior probabilities for TaPIN38
probability
transmembrane
inside
outside

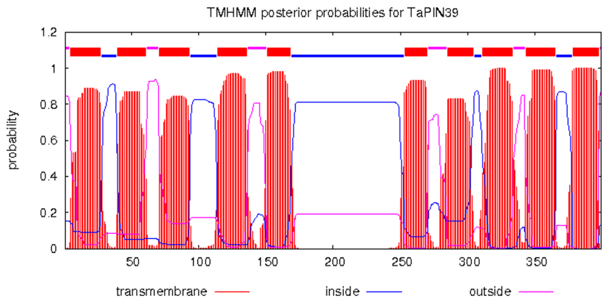
TMHMM posterior probabilities for TaPIN39
probability
transmembrane
inside
outside

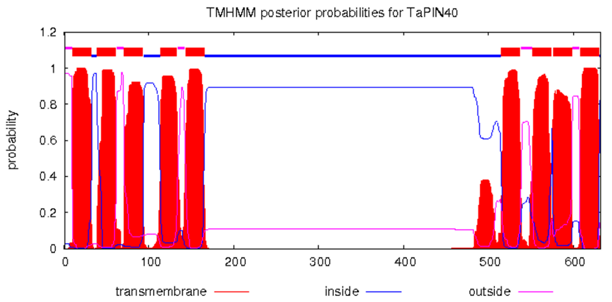
TMHMM posterior probabilities for TaPIN40
probability
transmembrane
inside
outside

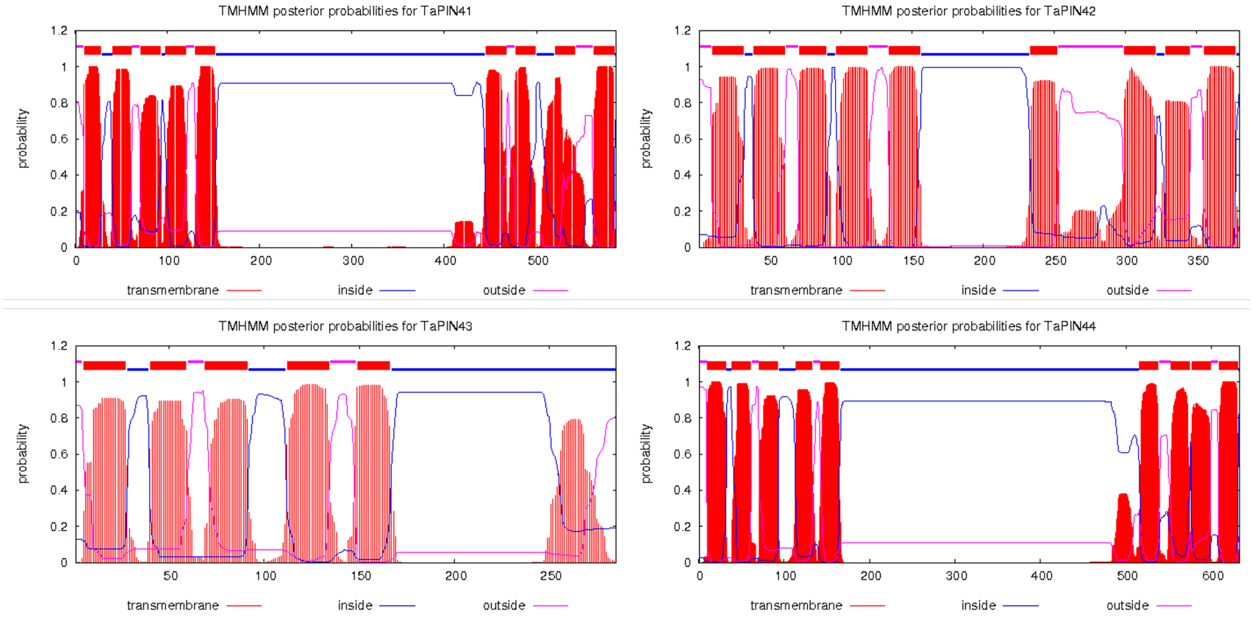

Figure S6. The Predicted Transmembrane Helices of the TaPIN Proteins. The transmembrane domains were identified using TMHMM2: (www.cbs.dtu.dk/services/TMHMM/) and SOSUI software tools (http://www.cbs.dtu.dk, http://harrier.nagahama-i-bio.ac.jp). The red peaks representing the predicted transmembrane domain and blue color line indicate the central hydrophobic loop of proteins.

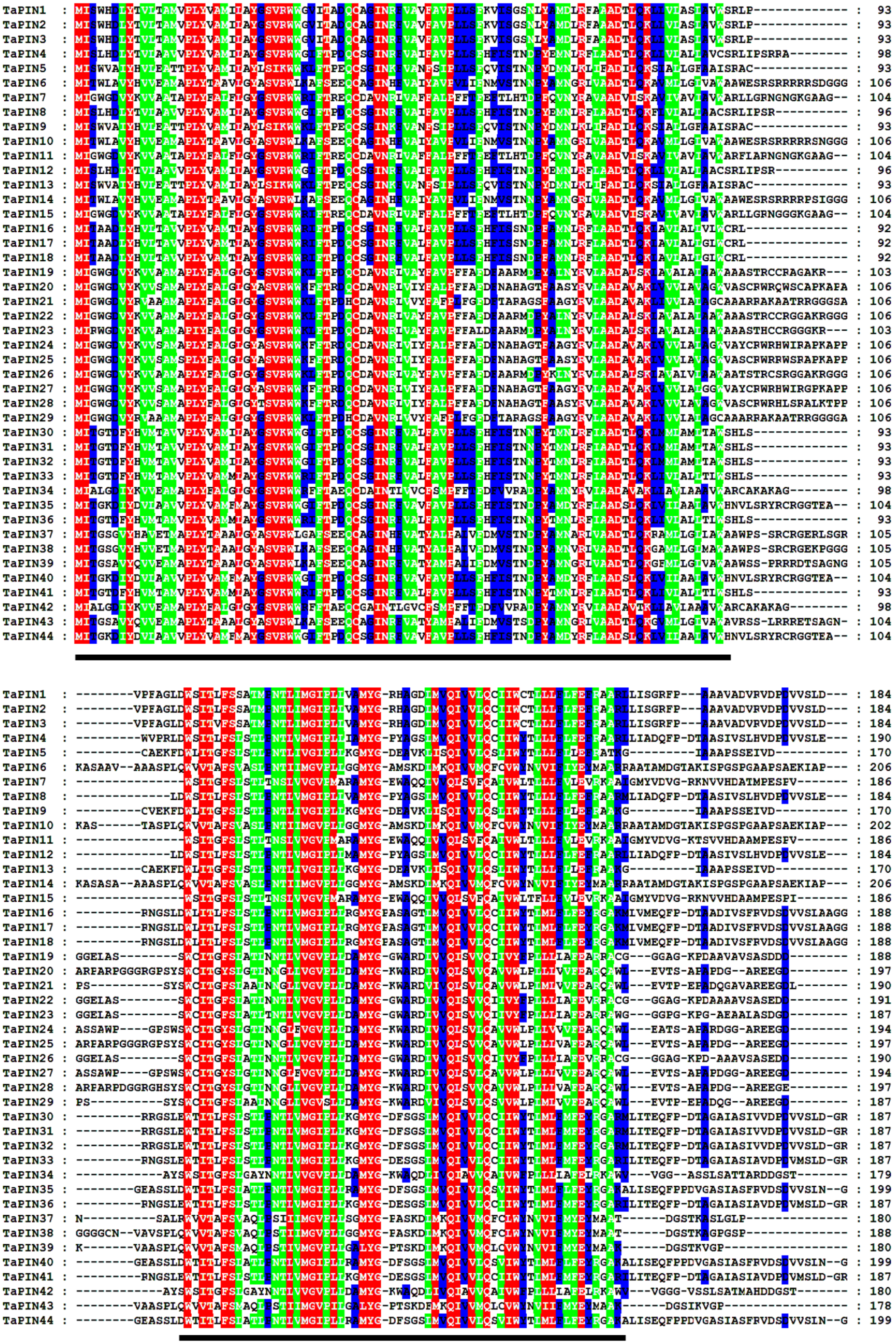

```
TaPIN1  : GSHAEAQAEVAPDGSMFVVVRRSAASLSRRSLINGAAAGMPS------FARESSVTGVEIFSVSSSRNHTPRGSSSFTHGDFSATTGGGGGAAAFALPP---PPPP : 281
TaPIN2  : GSHAEAQAEVAPDGSMFVVVRRSAASVSRRSLHNGAAAGMS-------PPRGSNLTGMEIYSVSSSRNHTPRGSSSFTHGDFSATTGGGG-GAAFALPP---PPPP : 279
TaPIN3  : GSHAEAQAEVAPDGSMFVVVRRSAASVSRRSLHNDAAAGMS-------PPRGSNLTGMEIYSVSSSRNHTPRGSSSFTHGDFSATTGGGG-AAAFALPP---PPP- : 278
TaPIN4  : GGHAEAESEVAADGRLHVTVSRSSVS-RRSTLL--------------VTPRPSNLTGAEIYSLSSSQNPTPRGSN-FNQADFFAVVGG----------------AP : 264
TaPIN5  : ------EEEAGTLG-----------------------------------------ATQQTYQEGQSKGVSARCSR------------------------------- : 198
TaPIN6  : ----NDEAVVAAKFAHEVTVNIEIT------------------------EVAFASTAQKDIADNTTTTVAKETSADA----------------------------- : 255
TaPIN7  : ------KADVEAAPTG--------AV---------------------------------AVLVVGGVEEVGGKPS------------------------------- : 214
TaPIN8  : GGRAETESEVAADGRLHVTVRRSSVS-RRSTLL--------------VTPRPSNLTGAEIYSLSSSQNPTPRGSN-FNQADFFAVVGG----------------AP : 258
TaPIN9  : ------EEEAGTLG-----------------------------------------ATQQTYQEGQSKGVSARCSR------------------------------- : 198
TaPIN10 : ----NDEAVVAAKFAHEVTVNIEIT------------------------EVAAASTAQKDIADNTTTTVAKETSTDA----------------------------- : 251
TaPIN11 : ------KADVEAAPT--------------------------------------------------GAEEVGGKPS------------------------------- : 205
TaPIN12 : GGRAETESEVAADGRLHVTVRRSSVS-RRSTLL--------------ATPRPSNLTGAEIYSLSSSQNPTPRGSN-FNQADFFAVVGG----------------AP : 258
TaPIN13 : ------EEEAGTLG-----------------------------------------ATQQTYQEGQSKGVSARCSR------------------------------- : 198
TaPIN14 : ----NDEAIVAAKFAHEVTVNIEIT------------------------EVAFASTVQKDIADNTTTTVAREMSTDA----------------------------- : 255
TaPIN15 : ------KADVEAAAGGG-------AV---------------------------------AVLVVGGAEEVGGKPS------------------------------- : 215
TaPIN16 : GADLQAEAEVGEDGRMFVTVRKSTS--SRSEAACSHSHSHSH--SQSMQPFVSNLSGVEIYSLQSSRNPTPRGSS-FNHAEFFNIVG-----------------GA : 272
TaPIN17 : GADLQAEAEVGEDGRMFVTVRKSTS--SRSEAACSHSHSHSH--SQSMQPFVSNLSGVEIYSLQSSRNPTPRGSS-FNHAEFFNIVG-----------------GA : 272
TaPIN18 : GADLQAEAEVGEDGRMFVTVRKSTS--SRSEAACSHSHSHSH--SQSMQPFVSNLSGVEIYSLQSSRNPTPRGSS-FNHAEFFNIVG-----------------GA : 272
TaPIN19 : -------VEDGG---------------------------------------------------AEGRRRREP---------------------------------- : 202
TaPIN20 : ------QAVPGSDDVDR------------------------------------------FAAAGDGRKTAATGWA------------------------------- : 224
TaPIN21 : ------QEAPGSDVHR-------------------------------------------FAAAGDGRKMAATGCA------------------------------- : 216
TaPIN22 : -------VEDGG---------------------------------------------------AEGRRRREP---------------------------------- : 205
TaPIN23 : -------VEDGG---------------------------------------------------AEDSRQRQP---------------------------------- : 201
TaPIN24 : ------QAALGSDD-------------------------------------------------GDGRKTATTGCA------------------------------- : 214
TaPIN25 : ------QAAPGSDDVDR------------------------------------------FAAAGEDRKMAATGWA------------------------------- : 224
TaPIN26 : -------VEDGG---------------------------------------------------AEGRRRRQP---------------------------------- : 204
TaPIN27 : ------QAALGSDD-------------------------------------------------GDGRKTAATGCA------------------------------- : 214
TaPIN28 : ------QAAPGSDDVDR------------------------------------------FAAAGDGRKTAATGWA------------------------------- : 224
TaPIN29 : ------QEAPGSDVHR-------------------------------------------FAASRDGRKTAATGCA------------------------------- : 213
TaPIN30 : SNAIETEAEVKEDGKIHVTVRRSSA--SRSDIYSRRSMGFS-----STTPRPSNLTNAEIYSLQSSRNPTPRGSS-FNHTDFYSMVGRSSNFGAADAYG--IRTGA : 283
TaPIN31 : SNAIETEAEVKEDGKIHVTVRRSSA--SRSDIYSRRSMGFS-----STTPRPSNLTNAEIYSLQSSRNPTPRGSS-FNHTDFYSMVGRSSNFGAADAYG--IRTGA : 283
TaPIN32 : SNAIETEAEVKEDGKIHVTVRRSSA--SRSDIYSRRSMGFS-----STTPRPSNLTNAEIYSLQSSRNPTPRGSS-FNHTDFYSMVGRSSNFGAADAYG--VRTGA : 283
TaPIN33 : RDMIETEAEVKEDGKIHVTVRRSNA--SRSDIYSRRSMGFS-----STTPRPSNLTNAEIYSLQSSRNPTPRGSS-FNHTDFYSMVGRSSNFAAGDAFGFVVRTGA : 285
TaPIN34 : ------TSDDDRAQHVE-------------------------------------------MDVEAAPPTGGRRIR------------------------------- : 205
TaPIN35 : REALHADAEVGRDGRVHVVIRRSASG-STTGGHGAGRSGIYRGASNAMTPRASNLTGVEIYSLQTSREPTPRQSS-FNQSDFYSMFNGSKLASPKGQPP--VAGTP : 301
TaPIN36 : RDMIETEAEVKEDGKIHVTVRRSNA--SRSDIYSRRSMGFS-----STTPRPSNLTNAEIYSLQSSRNPTPRGSS-FNHTDFYSMVGRSSNFAAGDAFGFVVRTGA : 285
TaPIN37 : ----EKRAIAAVERVHQVTVNVEST------------------------EHTRQQ-GMAVAGQQATAALTTEVCAGE----------------------------- : 228
TaPIN38 : ----EKRAIAAVERAHQVTVNVEST------------------------EHTRQQ-GMAVAGQQAAAALTTEVCAGE----------------------------- : 236
TaPIN39 : -------AFASPE-------NVEST------------------------EVQAAQQGMAAASQTTSTAVVGEASPTHGA--------------------------- : 221
TaPIN40 : REALHADAEVGRDGRVHVVIRRSASG-STTGGHGAGRSGIYRGASNAMTPRASNLTGVEIYSLQTSREPTPRQSS-FNQSDFYSMFNGSKLASPKGQPP--VAGGG : 301
TaPIN41 : RDMIETEAEVKEDGKIHVTVRRSNA--SRSDIYSRRSMGFS-----STTPRPSNLTNAEIYSLQSSRNPTPRGSS-FNHTDFYSMVGRSSNFAAGDAFGFVVRTGA : 285
TaPIN42 : ------SSDDDRARRVEFVSHSDVEV---------------------------------DLDVEAAPPTGGRRIR------------------------------- : 216
TaPIN43 : -------AFASPE-------NVGST------------------------EVAAAQPGMAVASQTTSTAVVGEVSPTHAA--------------------------- : 219
TaPIN44 : REALHADAEVGRDGRVHVVIRRSASG-STTGGHGAGRSGIYRGASNAMTPRASNLTGVEIYSLQTSREPTPRQSS-FNQSDFYSMFNGSKLASPKGQPP--VAGGG : 301
```

```
TaPIN1  : HGAVFASSFGAADLFSLHSSRQHTP-RPSASYDEHAPRGRSTAAVAFVEDPKD--NVHMFDWSSGASGASEVSG-LFVLRSS----AKESGRRRTPSDATSTNSDS : 379
TaPIN2  : HGAVFASSFGAADLFSLHSSRQHTP-RPSASYDEHAPRGRSAAAVAFVDDPKD--NVHMFDWSSGASGASEVSG-LFVFRSS----TKESGRRFAPSDTTSINSDS : 377
TaPIN3  : HGAVFASSFGAADLFSLHSSRQHTP-RPSASYDEHAPRGRSAAAVAFVEDPKD--NVHMFDWSSGASGASEVSG-LFVFRSS----AKESGRRFAPSDATSINSDS : 376
TaPIN4  : FARVDGSSFGASEHYSLQSSQGPTPRESNLDEHSAAPK--QAMTDAGAQHNHDAKELHMFVWSSSASFVSEVVSGLFVFSGG--TGAAHLDAAAKEIRMVVFADPP : 366
TaPIN5  : ------------------AFR------------------------------------------------------------------------------------- : 201
TaPIN6  : ---------------------------------------------------------------------------------------------------------- :   -
TaPIN7  : ---------------------------------------------------------------------------------------------------------- :   -
TaPIN8  : FARVGDSSFGASEHYSVQSSQRPTPRESNLEEHSAGPK--QAMTDAGAQHNHDAKELHMFVWSSSAFFVSEVVSGLFVFSGG--TDAAHLDAAAKEIRMVVFADPP : 360
TaPIN9  : ------------------AFR------------------------------------------------------------------------------------- : 201
TaPIN10 : ---------------------------------------------------------------------------------------------------------- :   -
TaPIN11 : ---------------------------------------------------------------------------------------------------------- :   -
TaPIN12 : FARVGGSSFGASEHYSLQSSQGPTPRESNLDEHSAGPK--HATTDAGAQHNHDAKELHMFVWSSSSSFVSEAVSGLFMFTGG--AGAAHLDAAAKEIRMVVFADPP : 360
TaPIN13 : ------------------AFR------------------------------------------------------------------------------------- : 201
TaPIN14 : ---------------------------------------------------------------------------------------------------------- :   -
TaPIN15 : ---------------------------------------------------------------------------------------------------------- :   -
TaPIN16 : K---GDEEKGSAGAGAGNGTGGHSP-QPLP-QAIAGKR-------------KD---LHMFVWSSSASFVSERAAGGAMHVFG----GGADHGDVL-AKGAQAYDEY : 352
TaPIN17 : K---GDEEKGSAGAG--NGTGGHSP-QPLP-QAIAGKR-------------KD---LHMFVWSSSASFVSERAAGGAMHVFG----GGADHGDVL-AKGAQAYDEY : 350
TaPIN18 : K---GDEEKGSAGAG--NGTGGHSP-QPLP-QAIAGKR-------------KD---LHMFVWSSSASFVSERAAGGAMHVFG----GGADHGDVL-AKGAQAYDEY : 350
TaPIN19 : ---------------------------------------------------------------------------------------------------------- :   -
TaPIN20 : ---------------------------------------------------------------------------------------------------------- :   -
TaPIN21 : ---------------------------------------------------------------------------------------------------------- :   -
TaPIN22 : ---------------------------------------------------------------------------------------------------------- :   -
TaPIN23 : ---------------------------------------------------------------------------------------------------------- :   -
TaPIN24 : ---------------------------------------------------------------------------------------------------------- :   -
TaPIN25 : ---------------------------------------------------------------------------------------------------------- :   -
TaPIN26 : ---------------------------------------------------------------------------------------------------------- :   -
TaPIN27 : ---------------------------------------------------------------------------------------------------------- :   -
TaPIN28 : ---------------------------------------------------------------------------------------------------------- :   -
TaPIN29 : ---------------------------------------------------------------------------------------------------------- :   -
TaPIN30 : TPRPSNYEEDAPKPKHFAPGAGHYP-AFNP-AVAAAPKGPKKFAANGQAKGED---LHMFVWSSSASFVSDVFGGGAPDYND----AAAAKSPRK-MDGAKERDDY : 379
TaPIN31 : TPRPSNYEEDAPKPKHFAPGAGHYP-AFNP-AVAAAPKGPKKFATNGQAKGED---LHMFVWSSSASFVSDVFGGGAPDYND----AAAAKSPRKSMDGAKEREDY : 380
TaPIN32 : TPRPSNYEEDAPKPKHFAPGAGHYP-AFNP-AVAAAPKGPKKFAANGQAKGED---LHMFVWSSSASFVSDVFGGGAPDYND----AAAAKSPRK-MDGAKERDDY : 379
TaPIN33 : TPRPSNYEEDKAGNNNNNSKYGQYP-AFNP-AMAAPQK-FAKKAANGQAKGEDGKDLHMFVWSSSASFVSDVFGNGTEAYND----AAAKDVRVA---AASPRKAD : 381
TaPIN34 : ---------------------------------------------------------------------------------------------------------- :   -
TaPIN35 : GARGQGLDEQVANKFKGGEAAAPYP-AFNPGMMMFAPR-KKELGGSNSNSNKE---LHMFVWSSSASFVSEANLFNAVNHAASTDFAAAPFAAATPRDGATPRGVS : 402
TaPIN36 : TPRPSNYEEDKAGNNNN-SKYGQYP-AFNP-AMAAPQK-FAKKAANGQAKGEDGKDLHMFVWSSSASFVSDVFGNGTEAYND----AAAKDVRVA---AASPRKAD : 380
TaPIN37 : ---------------------------------------------------------------------------------------------------------- :   -
TaPIN38 : ---------------------------------------------------------------------------------------------------------- :   -
TaPIN39 : ---------------------------------------------------------------------------------------------------------- :   -
TaPIN40 : GARGQGLDEQVANKFKGGEAAAPYP-AFNPGMMMFAPR-KKELGGSNSNSNKE---LHMFVWSSSASFVSEANLFNAVNHAASTDFAAAPFAAATPRDGATPRGVS : 402
TaPIN41 : TPRPSNYEEDKAGGNNN-SKYGQYP-AFNP-AMAAPQK-PSKKAANGQAKGEDGKDLHMFVWSSSASFVSDVFGNGTEAYND----AAAKDVRVA---AASPRKAD : 380
TaPIN42 : ---------------------------------------------------------------------------------------------------------- :   -
TaPIN43 : ---------------------------------------------------------------------------------------------------------- :   -
TaPIN44 : GARGQGLDEQVANKFKGGEAAAPYP-AFNPGMMMFAPRRKKELGGSNSNSNKE---LHMFVWSSSASFVSEANLFNAVNHAASTDFAAAPFAAATPRDGATPRGVS : 403
```

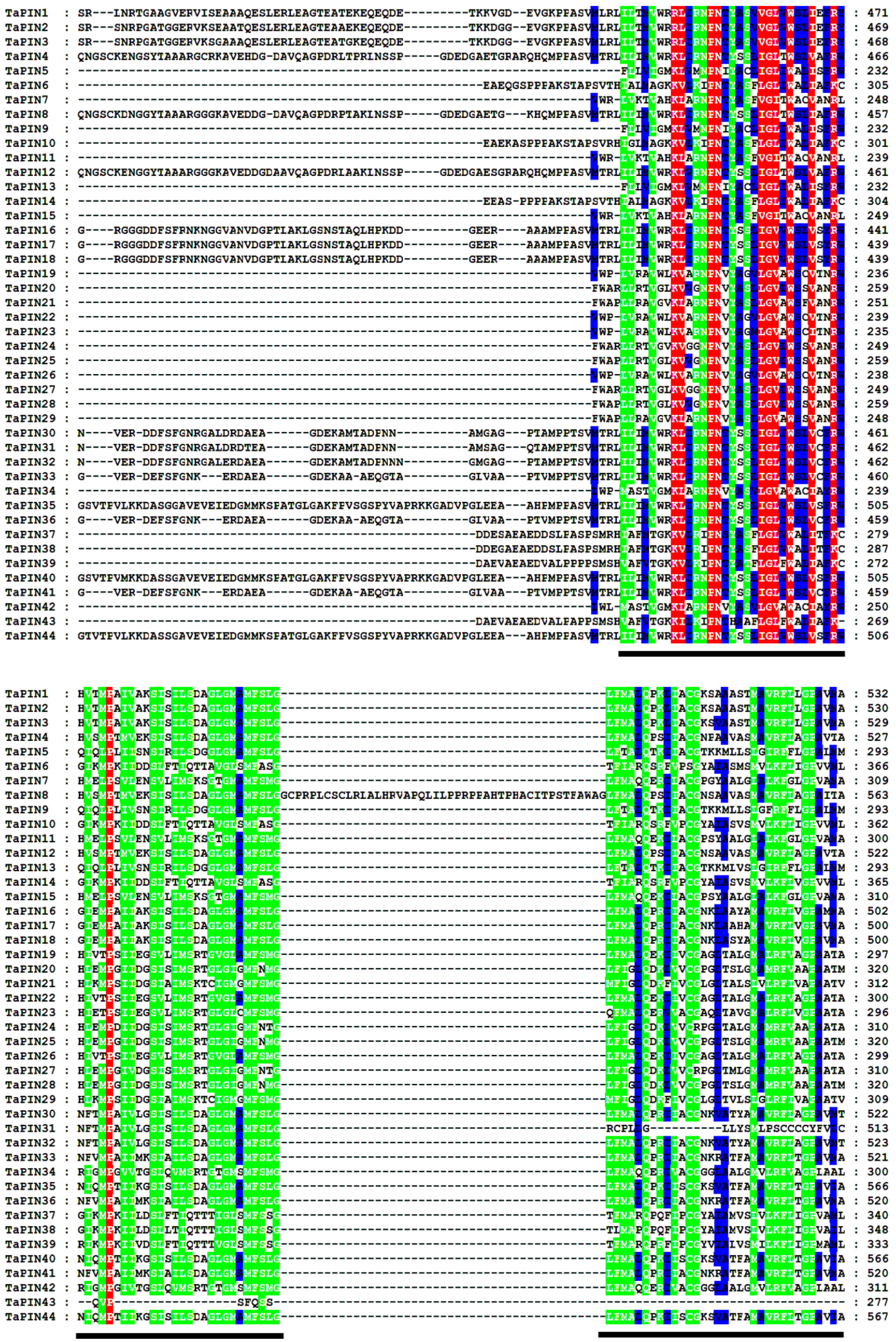
TaPIN1 : 471
TaPIN2 : 469
TaPIN3 : 468
TaPIN4 : 466
TaPIN5 : 232
TaPIN6 : 305
TaPIN7 : 248
TaPIN8 : 457
TaPIN9 : 232
TaPIN10 : 301
TaPIN11 : 239
TaPIN12 : 461
TaPIN13 : 232
TaPIN14 : 304
TaPIN15 : 249
TaPIN16 : 441
TaPIN17 : 439
TaPIN18 : 439
TaPIN19 : 236
TaPIN20 : 259
TaPIN21 : 251
TaPIN22 : 239
TaPIN23 : 235
TaPIN24 : 249
TaPIN25 : 259
TaPIN26 : 238
TaPIN27 : 249
TaPIN28 : 259
TaPIN29 : 248
TaPIN30 : 461
TaPIN31 : 462
TaPIN32 : 462
TaPIN33 : 460
TaPIN34 : 239
TaPIN35 : 505
TaPIN36 : 459
TaPIN37 : 279
TaPIN38 : 287
TaPIN39 : 272
TaPIN40 : 505
TaPIN41 : 459
TaPIN42 : 250
TaPIN43 : 269
TaPIN44 : 506
TaPIN1 : 532
TaPIN2 : 530
TaPIN3 : 529
TaPIN4 : 527
TaPIN5 : 293
TaPIN6 : 366
TaPIN7 : 309
TaPIN8 : 563
TaPIN9 : 293
TaPIN10 : 362
TaPIN11 : 300
TaPIN12 : 522
TaPIN13 : 293
TaPIN14 : 365
TaPIN15 : 310
TaPIN16 : 502
TaPIN17 : 500
TaPIN18 : 500
TaPIN19 : 297
TaPIN20 : 320
TaPIN21 : 312
TaPIN22 : 300
TaPIN23 : 296
TaPIN24 : 310
TaPIN25 : 320
TaPIN26 : 299
TaPIN27 : 310
TaPIN28 : 320
TaPIN29 : 309
TaPIN30 : 522
TaPIN31 : 513
TaPIN32 : 523
TaPIN33 : 521
TaPIN34 : 300
TaPIN35 : 566
TaPIN36 : 520
TaPIN37 : 340
TaPIN38 : 348
TaPIN39 : 333
TaPIN40 : 566
TaPIN41 : 520
TaPIN42 : 311
TaPIN43 : 277
TaPIN44 : 567

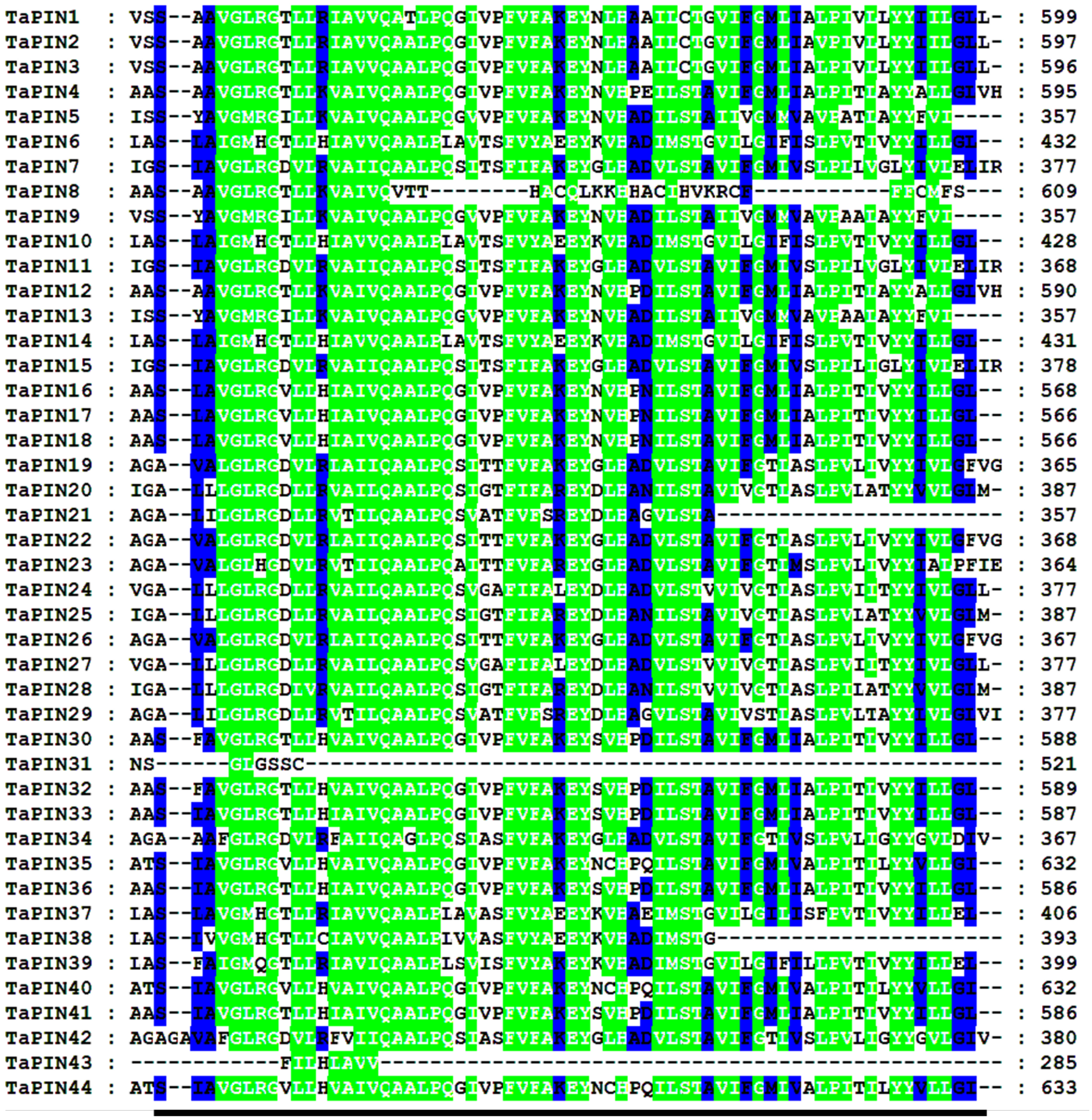

Figure S7. Alignment of the TaPINs protein sequences. The highly conserved regions of the TaPIN proteins were underlined with black color. Colored and shaded amino acids are chemically similar residues. Dashes indicate gaps introduced to maximize the alignment of homologous region.

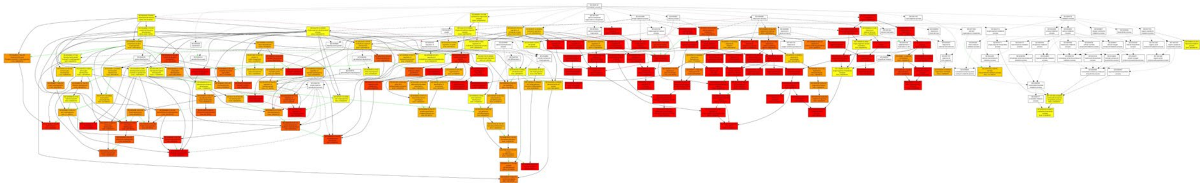

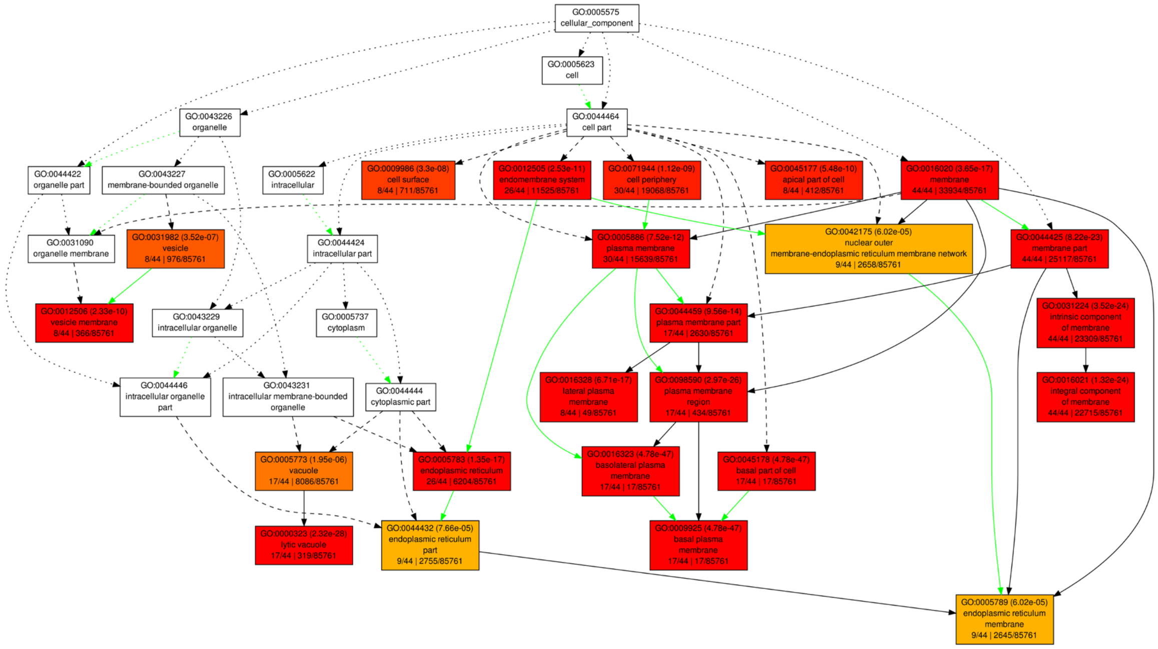

GO:0005575
cellular_component
GO:0005623
cell
GO:0043226
organelle
GO:0044464
cell part
GO:0044422
organelle part
GO:0043227
membrane-bounded organelle
GO:0005622
intracellular
GO:0009986 (3.3e-08)
cell surface
8/44 | 711/85761
GO:0012505 (2.53e-11)
endomembrane system
25/44 | 11525/85761
GO:0071944 (1.12e-09)
cell periphery
30/44 | 19068/85761
GO:0045177 (5.48e-10)
apical part of cell
8/44 | 412/85761
GO:0016020 (3.65e-17)
membrane
44/44 | 33934/85761
GO:0031090
organelle membrane
GO:0031982 (3.52e-07)
vesicle
8/44 | 976/85761
GO:0044424
intracellular part
GO:0005886 (7.52e-12)
plasma membrane
30/44 | 15639/85761
GO:0042175 (6.02e-05)
nuclear outer
membrane-endoplasmic reticulum membrane network
9/44 | 2658/85761
GO:0044425 (8.22e-23)
membrane part
44/44 | 25117/85761
GO:0012506 (2.33e-10)
vesicle membrane
6/44 | 366/85761
GO:0043229
intracellular organelle
GO:0005737
cytoplasm
GO:0044459 (9.56e-14)
plasma membrane part
17/44 | 2630/85761
GO:0031224 (3.52e-24)
intrinsic component
of membrane
44/44 | 23309/85761
GO:0044446
intracellular organelle
part
GO:0043231
intracellular membrane-bounded
organelle
GO:0044444
cytoplasmic part
GO:0016328 (6.71e-17)
lateral plasma
membrane
8/44 | 49/85761
GO:0098590 (2.97e-26)
plasma membrane
region
17/44 | 434/85761
GO:0016021 (1.32e-24)
integral component
of membrane
44/44 | 22715/85761
GO:0005773 (1.95e-06)
vacuole
17/44 | 8086/85761
GO:0005783 (1.35e-17)
endoplasmic reticulum
25/44 | 6204/85761
GO:0016323 (4.78e-47)
basolateral plasma
membrane
17/44 | 17/85761
GO:0045178 (4.78e-47)
basal part of cell
17/44 | 17/85761
GO:0000323 (2.32e-28)
lytic vacuole
17/44 | 319/85761
GO:0044432 (7.66e-05)
endoplasmic reticulum
part
9/44 | 2755/85761
GO:0009925 (4.78e-47)
basal plasma
membrane
17/44 | 17/85761
GO:0005789 (6.02e-05)
endoplasmic reticulum
membrane
9/44 | 2645/85761

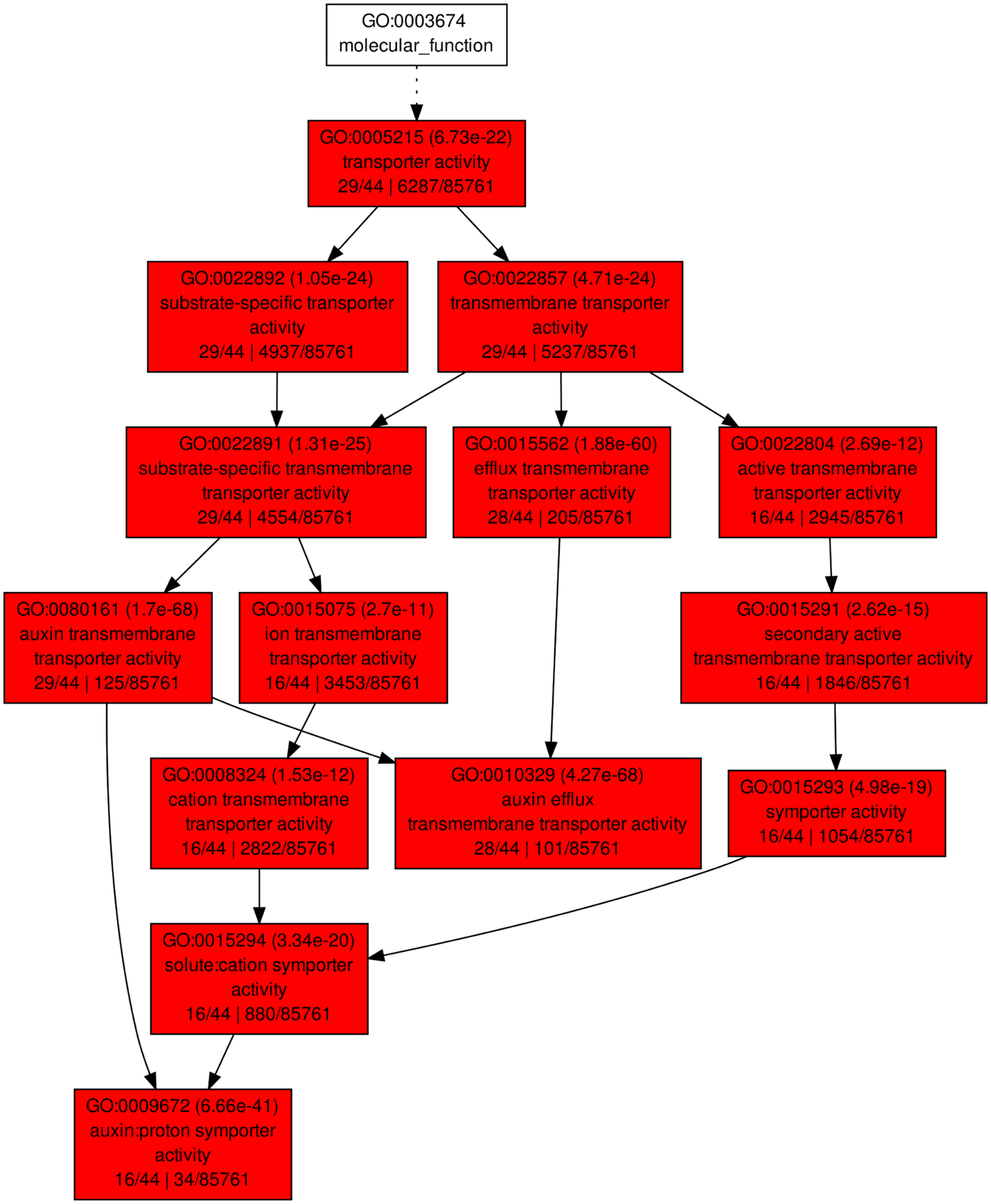

Figure S8. Gene ontology term distribution TaPIN gene family predicted using AgriGO **A.** Biological Process. **B.** Cellular component. **C.** Molecular function.

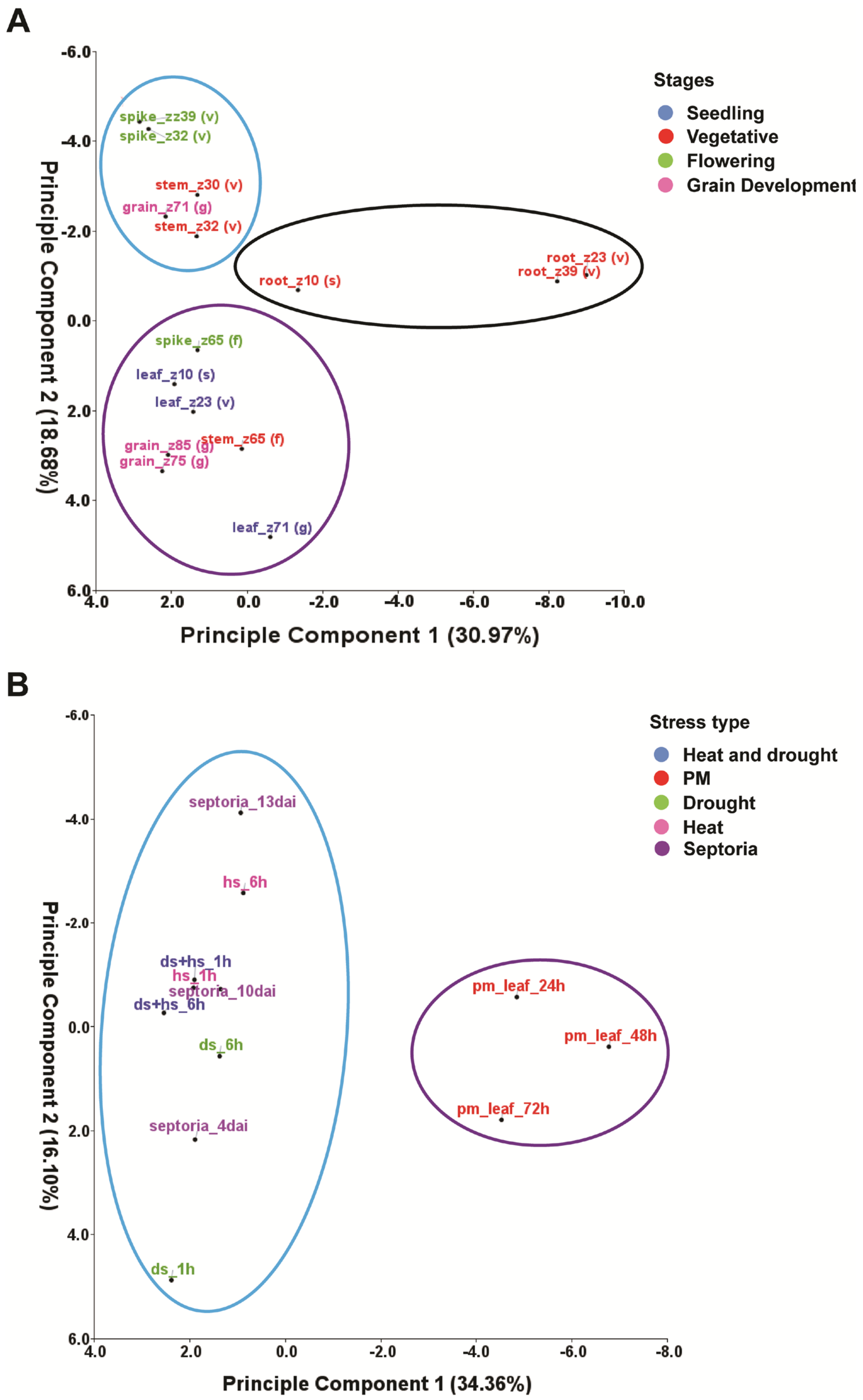

Figure S9: PCA plots displaying grouping of different (A) Developmental stages (B) Biotic and abiotic stress conditions based on the TaPIN expression pattern.

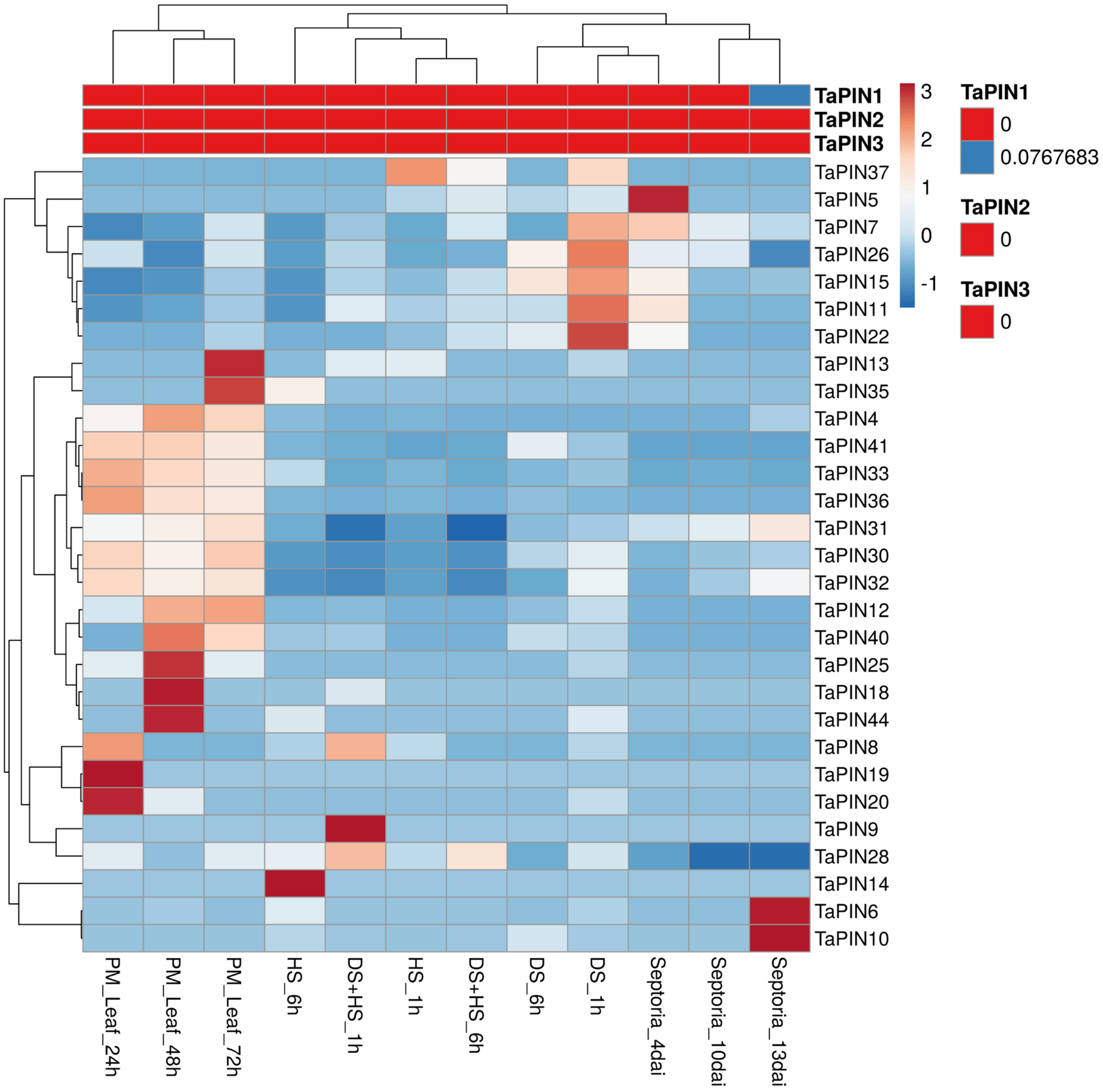

Figure S10: Heatmaps representing the expression pattern of TaPIN genes in different stress conditions. TPM values were directly used to construct the heatmaps. FPKM values were directly used to create the heatmaps. DS: Drought stress, HS: Heat stress, h: hour, d: days. PM: Powdery mildew.

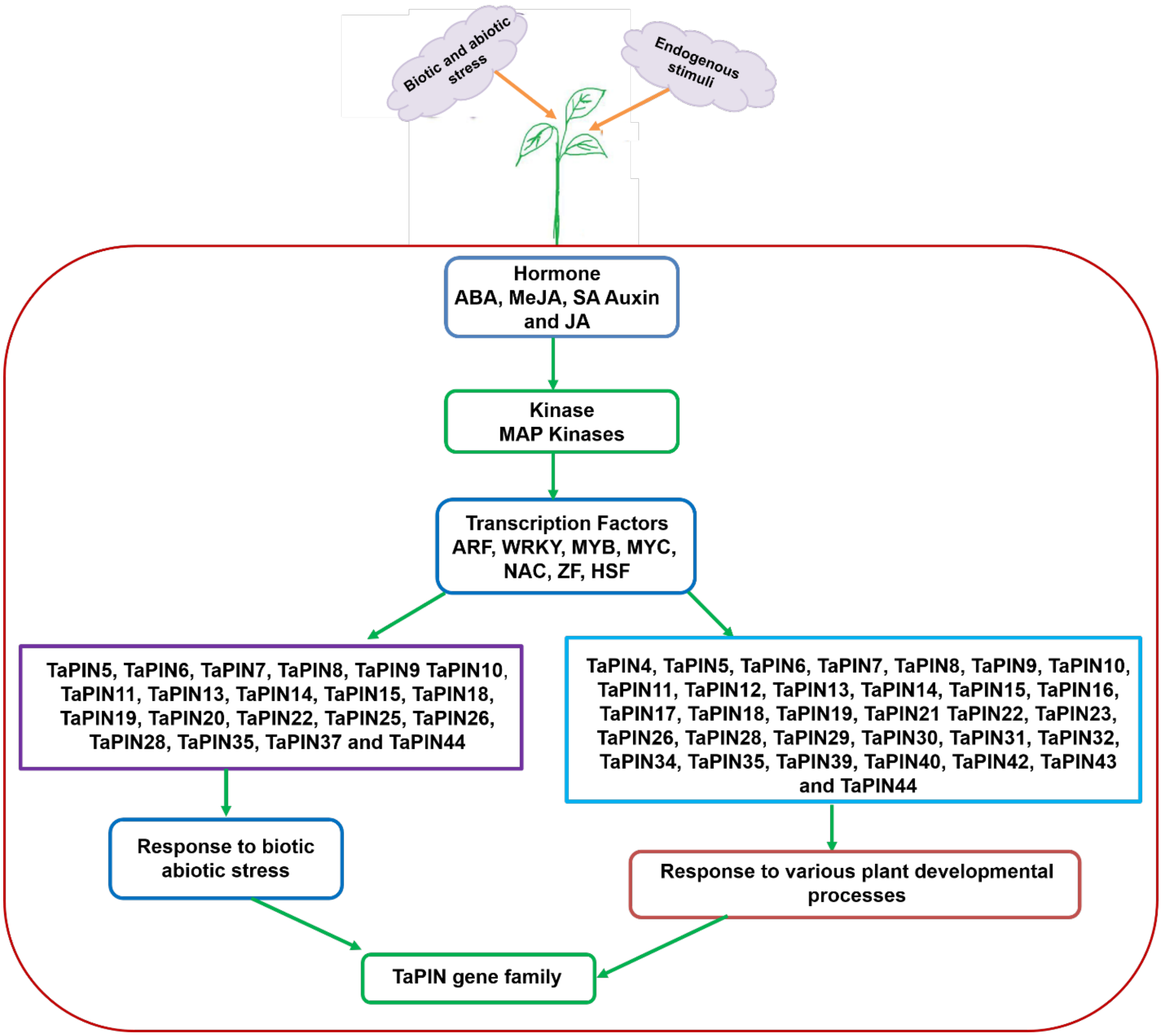

Figure S11. A possible function of TaPIN gene family in various plant developmental processes and diverse stress conditions. ABA: Abscisic acid; MeJA: Methyl jasmonate; SA: Salicylic acid; JA: Jasmonic acid; MAP: Mitogen-activated protein kinase; ARF: Auxin response factors; MYB: Myeloblastosis; NAC: No apical meristem; ZF: Zinc Finger and HSF: Heat shock factors.
